# Reparative Effects of Stem Cell Factor and Granulocyte Colony-Stimulating Factor in Aged APP/PS1 Mice

**DOI:** 10.14336/AD.2020.0201

**Published:** 2020-12-01

**Authors:** Xingzhi Guo, Yanying Liu, David Morgan, Li-Ru Zhao

**Affiliations:** ^1^Department of Neurosurgery, State University of New York Upstate Medical University, Syracuse, New York, 13210, USA; ^2^Translational Neuroscience, Michigan State University, College of Human Medicine, Grand Rapids, Michigan, 49503, USA

**Keywords:** Alzheimer’s disease, β-amyloid, granulocyte colony-stimulating factor, stem cell factor, microglia, neuroinflammation

## Abstract

Alzheimer’s disease (AD), characterized by the accumulation of β-amyloid (Aβ) plaques and tau neurofibrillary tangles in the brain, neuroinflammation and neurodegeneration, is the most common form of neurodegenerative disease among the elderly. No effective treatment is available now in restricting the pathological progression of AD. The aim of this study is to determine the therapeutic efficacy of stem cell factor (SCF) and granulocyte colony-stimulating factor (G-CSF) (SCF+G-CSF) in aged APPswe/PS1dE9 (APP/PS1) mice. SCF+G-CSF was subcutaneously injected for 12 days to 25-month-old male APP/PS1 mice. We observed that SCF+G-CSF treatment reduced the Aβ plaques in both the cortex and hippocampus. SCF+G-CSF treatment increased the association of TREM2^+^/Iba1^+^ cells with Aβ plaques and enhanced Aβ uptake by Iba1^+^ and CD68^+^cells in the brains of aged APP/PS1 mice. Importantly, cerebral expression area of P2RY12^+^and TMEM119^+^ homeostatic microglia and the branches of P2RY12^+^ homeostatic microglia were increased in the SCF+G-CSF-treated aged APP/PS1 mice. SCF+G-CSF treatment also decreased NOS-2 and increased IL-4 in the brains of aged APP/PS1 mice. Moreover, the loss of MAP2^+^dendrites and PSD-95^+^post-synapses and the accumulation of aggregated tau in the brains of aged APP/PS1 mice were ameliorated by SCF+G-CSF treatment. Furthermore, the density of P2RY12^+^ microglia was negatively correlated with Aβ deposits, but positively correlated with the densities of MAP2^+^ dendrites and PSD-95^+^ puncta in the brains of aged APP/PS1 mice. These findings reveal the therapeutic potential of SCF+G-CSF treatment in ameliorating AD pathology at the late stage.

Alzheimer's disease (AD) is the most common neurodegenerative disease in the elderly, which leads to huge economic costs and social burden worldwide [1]. In the United States, an estimated 5.8 million Americans were living with AD in 2019, and 97% of AD individuals are age 65 and older [2].

The pathological hallmarks of AD are characterized by the presence of amyloid beta (Aβ) plaques, intracellular neurofibrillary tangles, and neuro-inflammation [3, 4]. The Aβ, a peptide with 40-42 amino acids, is derived from the amyloid precursor protein (APP) through sequentially proteolytic cleavages by the β-secretase and γ-secretase [5]. Overproduction or insufficient clearance of Aβ leads to Aβ aggregation, and the pathological accumulation of oligomeric Aβ is crucially involved in neuronal dysfunction, neuronal network destabilization and cognitive impairment [6]. Moreover, intracerebral injection of aggregated Aβ _(25-35)_ chronically activates microglia, resulting in persistent neuroinflammation [7]. It has been reported that inhibiting Aβ plaque formation protects against dendritic spine loss and prevents cognitive deficits in an APP mouse model of AD [8]. Therefore, to prevent Aβ accumulation or promote Aβ removal has been deemed the main therapeutic target for AD [9-11]. Over the past decades, tremendous efforts have been made to develop treatment for AD, while effective medications have not yet been available to stop, delay or reverse the pathological progression of AD [11]. Anti-Aβ immunotherapy with anti-Aβ antibodies has been thought to be a hopeful therapeutic strategy for AD [12-16]. A long-term study of monthly administration of a human monoclonal antibody, aducanumab, to mild AD patients for one year has shown reduced brain Aβ and slowed cognitive decline [12], leading to more recent AD clinical trials. However, lack of efficacy or the adverse effects (e.g. amyloid-related imaging abnormalities) found in clinical trials, and considerable costs of long-term treatment limit the usage of this therapy in AD [12, 16-19]. Therefore, to develop new effective and safe pharmaceutical approaches is an urgent need for preventing or delaying the progression of AD.

Stem cell factor (SCF) and granulocyte colony-stimulating factor (G-CSF) were originally demonstrated as essential hematopoietic growth factors regulating the proliferation, differentiation and mobilization of hematopoietic stem cells (HSCs) [20, 21]. SCF in combination with G-CSF (SCF+G-CSF) synergistically enhances the proliferation, differentiation, and survival of HSCs/hematopoietic progenitor cells (HPCs) [22], and synergistically mobilizes HSCs/HPCs into the blood stream [23-25]. The biologic synergy between SCF and G-CSF has also been demonstrated in the central nervous system. SCF+G-CSF synergistically promotes neurite outgrowth and neural network structural formation of cultured cortical neurons through PI3K/AKT/NF-kB/BDNF pathway [26]. SCF+G-CSF treatment in the chronic phase of experimental stroke leads to stable and long-term improvements in somatosensory motor function as compared to SCF and G-CSF alone treatments [27], and enhances neural network regrowth and synaptogenesis [28, 29].

In clinical studies, decreased levels of SCF and G-CSF in the plasma have been observed in AD patients [30, 31]. It remains to be determined, however, whether exogenous administration of SCF and G-CSF could ameliorate pathological severity in AD. In our earlier study, we have revealed that systemic administration of SCF+G-CSF in middle aged (9 months) APP/PS1 mice results in long-term (9 months) reductions in Aβ deposits [32]. Since AD predominantly affects elderly people, it is highly important to determine the efficacy of SCF+G-CSF treatment in Aβ removal, neuroinflammatory modulation, and rebuilding neuronal connections in aged APP/PS1 mice.

In the present study, 25-month-old APP/PS1 mice received 12-days of SCF+G-CSF injections. As an initial study exploring pharmaceutical strategy for AD at such an old age, we have demonstrated that SCF+G-CSF treatment reduces Aβ deposits, ameliorates neuroinflammation, and increases the densities of dendrites and synapses in the brains of the very old APP/PS1 mice.

## MATERIALS AND METHODS

### Animals

The experiments have been carried out in accordance with the National Institutes of Health Guidelines for the Care and Use of Laboratory Animals in the United States. All experimental procedures have been approved by the Animal Care and Use Committees of State University of New York Upstate Medical University (IACUC#369).

APPswe/PS1dE9 (APP/PS1) transgenic mice co-expressing Swedish double mutations (K595N/M596L) of amyloid precursor protein (APP) together with mutant human presenilin protein 1 (PS1) carrying the exon-9-deleted variant (PS1-dE9)[33] were used in this study (stock# 34832, The Jackson Laboratory, Bar Harbor, Maine, USA). The APP/PS1 mice with C57BL/6J genetic background develop Aβ deposits in the brain at 6-7 months of age [34]. Mice were housed in a standard laboratory environment (12 h light/12 h dark regime at 22 °C) and were given free access to food and water.

### Experimental Design

**Figure 1.**
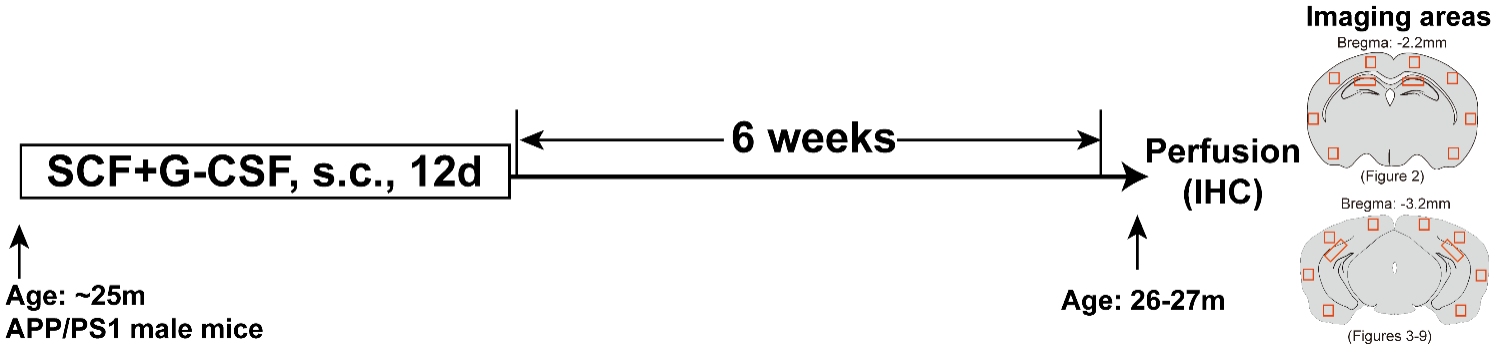
Experimental flowchart and imaging areas in the brain sections. Mouse brain diagrams illustrate the brain regions and the areas for imaging in the cortex and hippocampus. s.c., subcutaneously; IHC, immunohistochemistry.

A total of 20 male APP/PS1 mice were prepared for this study (n=10/group). At the age of ~25 months, nine of them remained. These aged APP/PS1 mice and five age-matched C57BL/6J wild type (WT) mice (The Jackson Laboratory, Bar Harbor, Maine, USA) were used in this study. The APP/PS1 mice were randomly divided into two groups and subcutaneously injected with recombinant mouse SCF (200 µg/kg/day; PeproTech, Rocky Hill, NJ, USA) and recombinant human G-CSF (50 µg/kg/day; Amgen, Thousand Oaks, CA, USA) (SCF+G-CSF) (n =4) or vehicle solution (saline and 5% dextrose) (n =5) once a day for 12 days. Recombinant SCF and G-CSF have been shown to pass through the blood-brain barrier in intact animals [35]. The doses [36, 37] and treatment duration [32] for SCF+G-CSF intervention were selected in the present study based on the effective paradigm demonstrated in our earlier studies. Six weeks after injection of SCF+G-CSF or vehicle, all the APP/PS1 and WT mice were sacrificed, and the brains were collected for immunohistochemistry/histochemistry (Fig. 1). We chose to examine the effects of treatment at six weeks post-treatment because our previous study in aged mice with chronic stroke has shown increased dendrites and synapses in the cortex six weeks after SCF+G-CSF treatment [28].

### Immunohistochemistry and histochemistry

Mice were transcardially perfused with phosphate buffered saline (PBS) followed by 4% buffered formaldehyde (Sigma-Aldrich, St. Louis, MO, USA). The brains were removed, placed in 4% buffered formal-dehyde overnight at 4°C, and then cryoprotected by immersion in 30% sucrose. Coronal brain sections (30μm thick) were cut with a microtome (Leica SM 2000R, Nussloch, Germany). Two or three brain sections from each brain were processed for immunohistochemistry or histochemistry. The free-floating method was used for immunohistochemistry. Briefly, to minimize endogenous mouse IgG background staining when using mouse primary antibodies, sections were incubated with mouse on mouse (MOM) solution (Vector Laboratories, Burlingame, CA, USA) for 1 h at room temperature. After being washed with PBS (3 × 5 min), nonspecific binding was blocked by 10% normal donkey serum in 1% bovine serum albumin (IgG-free, protease free, Jackson ImmunoResearch Labs, West Grove, PA, USA) and 0.25% Triton X-100 (Sigma-Aldrich, St. Louis, MO, USA) for 1 h at room temperature. Brain sections were then incubated with primary antibodies overnight at 4°C. The primary antibodies include mouse anti-β-amyloid (1:1000) (clone 4G8, Biolegend, Dedham, MA, USA, #800701), mouse anti-β-amyloid (1:1000) (clone 6E10, Biolegend, Dedham, MA, USA, #803004), rabbit anti-Iba1 (Ionized calcium binding adaptor molecule 1) (1:400, Wako, Osaka, Japan, #019-19741), goat anti-Iba1 (1:400, Novus Biologicals, CO, USA, #NB100-1028), rabbit anti-P2RY12 (purinergic receptor P2Y, G-protein coupled, 12) (1:500, Brigham and Women’s Hospital, MA, USA), rabbit anti-MAP2 (microtubule associated protein 2) (1:600, Millipore Corporation, MA, USA, #AB5622), mouse anti-PSD-95 (postsynaptic density protein 95) (1:500, Novus Biologicals, CO, USA, #NBP2-12872), sheep anti-TREM2 (triggering receptor expressed on myeloid cells-2) (1:300, R&D Systems, Minneapolis, USA, #AF1729), NOS2 (1:100, Millipore Corporation, MA, USA, #AB5382), IL-4 (1:50, Thermo Fisher Scientific, Waltham, MA, USA, # PA5-25165), NeuN (1:500, Millipore Corporation, MA, USA, #MAB377), GFAP (1:500, Sigma-Aldrich, St. Louis, MO, USA, #G3893), CD68 (1:300, Bio-Rad, Hercules, CA, USA, #MCA1957GA), TMEM119 (1:1000, Abcam, Cambridge, MA, #ab209064), and AT8 (1:400, Thermo Fisher Scientific, Waltham, MA, USA, #MN1020). After being washed with PBS (3 × 10 min), the sections were incubated with Alexa Fluor 488-, 594- or 647-conjugated donkey anti-mouse, rabbit, goat or sheep secondary antibodies (Thermo Fisher Scientific, Waltham, MA, USA) for 2 h at room temperature in the dark. After being washed with PBS (3 × 10 min), sections were mounted with Vectashield antifade mounting medium containing 4', 6-diamidino-2-phenylindole (DAPI) (Vector Laboratories, Burlingame, CA, USA, #H-1500).

For X-34 (Sigma-Aldrich, St. Louis, MO, USA) and Collagen-IV staining, Tris-EDTA Buffer (10mM Tris Base, 1mM EDTA Solution, pH 9.0) was used for antigen retrieval before staining. Briefly, after being washed with PBS for 5 min, the sections were put into pre-heated antigen retrieval buffer (500 ml) at 90°C for 10 min and then cooled to room temperature. After PBS washing (3 × 5min), the sections were rinsed with ddH_2_O for 2 min. The sections were then put into 100μM X-34 working solution (pH=10) for 10 min at room temperature in the dark. After being washed with ddH_2_O (5 × 2 min), the sections were differentiated for 2 min with 0.2% NaOH. After PBS washing (2 × 5 min), the sections were then incubated with Col-IV antibody (rabbit anti-Col-IV, 1:300, Abcam, Cambridge, MA, #ab6586) overnight at 4°C and incubated with Alexa Fluor 594-conjugated donkey anti-rabbit for 2h at room temperature following the standard immunohistochemistry protocol described above. Finally, the sections were mounted with Vectashield antifade mounting medium without DAPI (Vector Laboratories, Burlingame, CA, USA, #H-1700). Brain sections with the omission of primary antibodies served as negative controls (Supplementary Fig. 1).

### Quantification of immunohistochemistry and histo-chemistry

To determine the Aβ deposition in the brain, images were obtained using a Zeiss-inverted microscope (AxioVision 4.8, Carl Zeiss AG, Gottingen, Germany) and quantified using ImageJ software. Images were taken from eight selected areas in the cortex (bregma, -1.2 mm) and 6 selected areas in the hippocampus (bregma, -2.2 mm) of each brain section using a 20× or 40× objective lens (Fig. 1). Plaque number, plaque area and average Aβ size were calculated in 20× images using the “analyze particles plugin” of ImageJ. The Aβ area fraction was determined by dividing the total plaque area by the area of the microscopic field.

For the rest of immunohistochemistry quantification, four random areas in the cortex and 1 × 3 tile scan areas in the CA1 region (bregma, -3.2 mm) of each hemisphere were selected to capture images from each brain section using a confocal microscope (Zeiss LSM780) with a 20× or 40× objective lens (Fig. 1). In the analysis of Aβ phagocytosis by Iba1 positive cells, images (12 z-stacks with 1μm intervals, 1024 × 1024 pixels) were taken using the Zeiss LSM780 confocal microscope. The colocalization of Iba1 positive cells and 4G8 positive Aβ was quantified by using the 3D plugin in ImageJ. The volume of 4G8^+^senile plaques within Iba1^+^ cells surrounding the senile plaques in each image field was calculated. The Aβ uptake volume was determined by dividing the Iba1^+^/4G8^+^colocalization volume by the total Iba1^+^ cell volume. For quantification of Iba1, TREM2, CD68, P2RY12, TMEM119, AT8, NOS-2 and IL-4 immunopositive staining, images (12 z-stacks with 1μm intervals, 1024 × 1024 pixels) were taken with the Zeiss LSM780 confocal microscope using the 40× lens. After the images were stacked with the maximum approach, the immunopositive staining of Iba1, TREM2, CD68, P2RY12, TMEM119, AT8, NOS-2 and IL-4 was quantified by dividing the total immunopositive staining area by the area of the image field using ImageJ.

For quantification of MAP2 staining, images were captured with the Zeiss LSM780 confocal microscope using the tile scan feature with an average grid size of 3 × 5 taken with the 40× objective at a resolution of 1024 × 1024 pixels. The MAP2^+^ immunostaining in the cortex and CA1 was determined by dividing the MAP2 positive area by the area of the image field of the entire cortex or selected CA1 region using Image J. For quantification of PSD-95 staining, images (1024 × 1024 pixels) were taken by using the Zeiss LSM780 confocal microscope with the 40× lens. The number of PSD-95-labeled puncta in the cortex and CA1 was counted automatically with a threshold of 20 pixels in ImageJ.

For quantification of X-34^+^ Aβ deposition and Col-IV^+^ blood vessels, images were taken with the Zeiss LSM780 confocal microscope using the 20× lens. The X-34 and Col-IV positive staining were quantified by dividing X-34 and Col-IV positive area by the area of the image fields in the cortex or CA1 using ImageJ.

For all image analysis, the background fluorescence of secondary antibodies was established as a threshold to be subtracted from the positive staining during data analysis using ImageJ.

### Statistical analysis

Using Prism 6.0 software, comparisons among three groups were analyzed by one-way ANOVA followed by Fisher's LSD test, and the comparisons between two groups were analyzed by unpaired student’s t-test. Pearson correlation analysis was used to evaluate the correlative association between X-34^+^ Aβ deposition and P2RY12^+^ cells, between P2RY12^+^ cells and MAP2^+^ dendrites, and between P2RY12^+^ cells and PSD-95^+^ synapses. All data are expressed as mean ± S.E.M. Statistical significance was considered p<0.05.

## RESULTS

### SCF plus G-CSF treatment reduces β-amyloid deposition in aged APP/PS1 mice

**Figure 2.**
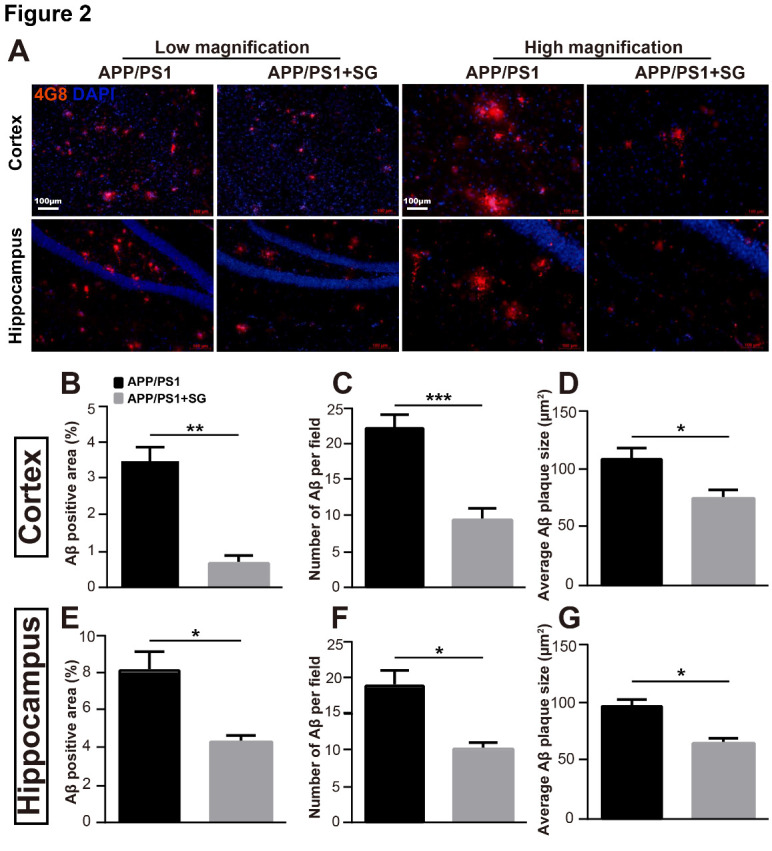
SCF+G-CSF treatment reduces Aβ deposits in the brains of aged APP/PS1 mice. (A) Representative images of 4G8^+^ (red) Aβ deposits in the cortex and hippocampus with both low magnification and high magnification. (B-D) Quantification data show the percentage of 4G8^+^ Aβ area (B), the number of 4G8^+^ Aβ plaques (C) and the average size of 4G8^+^ Aβ plaques (D) in the cortex of aged APP/PS1 male mice treated with or without SCF+G-CSF. (E-G) Quantification data illustrate the percentage of 4G8^+^ Aβ area (E), the number of 4G8^+^ Aβ plaques (F) and the average size of 4G8^+^ Aβ plaques (G) in the hippocampus of aged APP/PS1 mice treated with or without SCF+G-CSF. Blue: Nuclear counterstaining by 4',6-diamidino-2-phenylindole (DAPI). SG: SCF+G-CSF. N=4-5. Mean ± SEM. *p<0.05, **p<0.01, ***p<0.001 by Student’s t-test.

To determine the effects of SCF+G-CSF treatment on Aβ removal in aged male APP/PS1 mice, immune-histochemistry was performed to determine 4G8 positive compact fibrillar Aβ and diffuse Aβ [38] in the cortex and hippocampus. We observed that there were numerous 4G8^+^Aβ deposits in the cortex and hippocampus of APP/PS1 mice, while SCF+G-CSF treatment significantly reduced the percentage area of 4G8^+^Aβ deposits in both the cortex (p<0.01) and hippocampus (p<0.05) (Fig. 2A, B, E). In addition, the number of 4G8^+^Aβ plaques in the cortex was significantly decreased in the SCF+G-CSF-treated APP/PS1 mice as compared to the vehicle controls (p<0.001) (Fig. 2C). A similar result was also found in the hippocampus (vehicle control vs. SCF+G-CSF, p<0.05) (Fig. 2F). Moreover, the SCF+G-CSF treatment also significantly reduced the average size of 4G8^+^Aβ plaques in the cortex and hippocampus (p<0.05) (Fig. 2D, G). These findings suggest that SCF+G-CSF treatment in aged APP/PS1 mice reduces the load of diffuse Aβ and compact fibrillar Aβ in the cortex and hippocampus.

**Figure 3.**
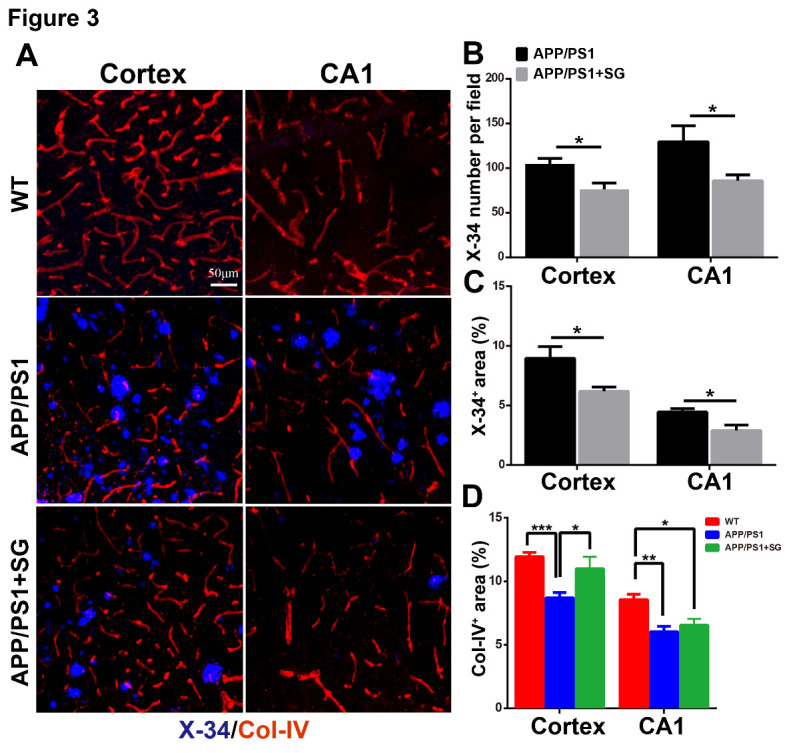
SCF+G-CSF treatment decreases fibrillar Aβ deposits and increases blood vessel density in the brains of aged APP/PS1 mice. (A) Representative confocal images show X-34^+^ fibrillar Aβ plaques (blue) and collagen IV (Col-IV) immunopositive blood vessels (red) in the cortex and hippocampal CA1 region of aged APP/PS1 mice and age-matched wild type (WT) mice. (B and C) Quantification data show the number of X-34^+^ fibrillar Aβ plaques (B) and the percentage of X-34^+^ fibrillar Aβ area (C) in the cortex and hippocampal CA1 of aged APP/PS1 mice. (D) Quantification data show the percentage of Col-IV^+^ area in the cortex and hippocampal CA1 of aged APP/PS1 mice and age-matched WT mice. N=4-5. Mean ± SEM. *p<0.05, **p<0.01, ***p<0.001 by Student’s t-test (B, C) or one-way ANOVA followed by Fisher’s LSD *post hoc* test.

Fibrillar Aβ is the primary constituent of amyloid plaques in the brain of AD [39, 40]. X-34 staining detects the compact fibrillar Aβ [41]. To further confirm the contribution of SCF+G-CSF treatment in Aβ clearance, we utilized X-34 staining to assess the compact fibrillar Aβ plaques in the brains of APP/PS1 mice. We observed that the SCF+G-CSF-treated APP/PS1 mice showed significant reductions in the number (Fig. 3A and B) and percentage areas (Fig. 3C) of X-34 positive plaques in both the cortex and hippocampal CA1 region (p<0.05). This observation indicates that systemic administration of SCF+G-CSF in aged APP/PS1 mice leads to reduction of compact fibrillar Aβ in the cortex and hippocampus.

Since vascular impairment has been shown to be involved in Aβ accumulation and AD progression [42], we then examined the vascular effects of SCF + G-CSF treatment through the analysis of vascular density in the brains of aged APP/PS1 mice. The data showed that the vascular density was decreased in both the cortex (p<0.001) and hippocampal CA1 (p<0.01) of aged APP/PS1 mice as compared to the WT controls (Fig. 3D). SCF+G-CSF treatment significantly reversed the vascular loss in the cortex (p<0.05) of aged APP/PS1 mice, while the treatment did not affect the blood vessel loss in the CA1 region (Fig. 3D).

Altogether, our findings suggest that SCF+G-CSF treatment in aged male APP/PS1 mice leads to remarkable reductions in cerebral Aβ load, which may be the result of increased removal of Aβ deposits in the brain by SCF+G-CSF intervention. The SCF+G-CSF-reduced Aβ load may be associated with, but not dependent on, its effects on maintaining vasculature in the brains of aged APP/PS1 mice.

**Figure 4.**
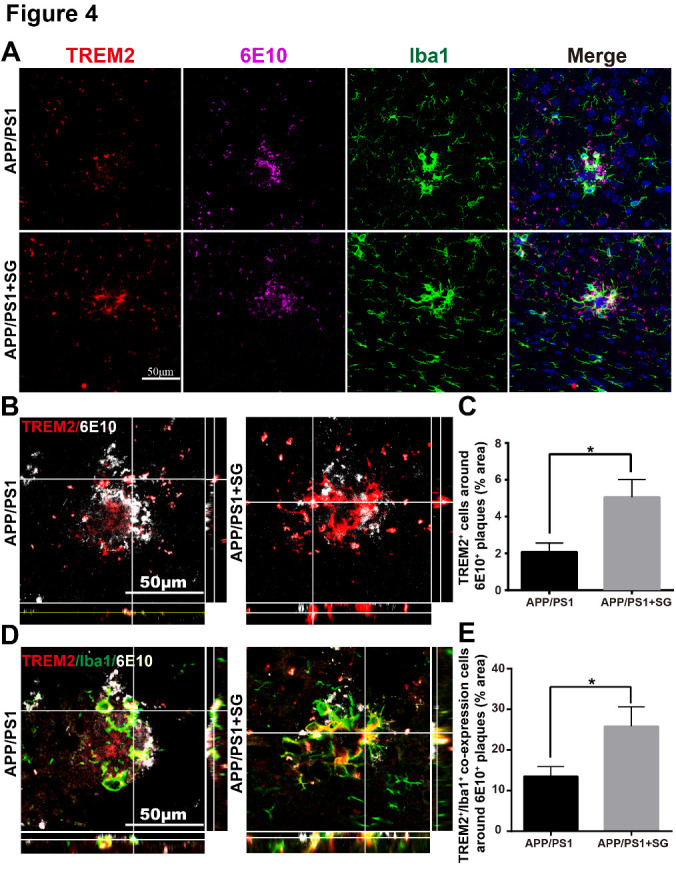
SCF+G-CSF treatment increases TREM2 expression in the Iba1^+^ microglia/macrophages surrounding the 6E10^+^ senile plaques. (A) Representative confocal images of TREM2 (red), 6E10 (purple) and Iba1 (green) triple immunofluorescence staining in the brains of aged APP/PS1 mice. Blue: Nuclear counterstaining by DAPI. (B) Representative orthographic view of z-stack images (12 z-stacks with 1μm intervals) illustrates the location and interaction of TREM2 ^+^ cells (red) and 6E10^+^ Aβ plaques (white) in the brains of aged APP/PS1 mice. (C) Quantification data show the percentage of TREM2^+^ area surrounding the 6E10^+^ Aβ plaques (within 10μm from the border of the Aβ plaques) in the brains of aged APP/PS1 mice with or without SCF+G-CSF treatment. (D) Representative orthographic view of z-stack images (12 z-stacks with 1μm intervals) displays the location and interaction of TREM2^+^/Iba1^+^ co-expressing cells (yellow) and 6E10^+^ Aβ plaques (white) in the brains of APP/PS1 mice. (E) Quantification data show the percentage of TREM2^+^/Iba1^+^ co-expression area in the total of Iba1^+^ area in the vicinity of 6E10^+^ Aβ plaques in the brains of aged APP/PS1 mice with or without SCF+G-CSF treatment. N=4-5. Mean ± SEM. * p<0.05 by Student’s t-test.

### SCF plus G-CSF treatment increases TREM2 expression in the Iba1^+^ cells around the plaques in aged APP/PS1 mice

Recent studies have demonstrated a vital role of TREM2 in mediating the phagocytosis of Aβ by microglial cells [43, 44]. To evaluate whether TREM2 was involved in the increased clearance of Aβ by Iba1^+^cells, triple immunofluorescence staining of Iba1, TREM2 and 6E10 was performed in brain sections of APP/PS1 mice. We observed that SCF+G-CSF treatment significantly increased the TREM2 expression around the 6E10^+^Aβ plaques in the brains of APP/PS1 mice as compared to the vehicle controls (p<0.05) (Fig. 4A-C) (Supplementary Fig. 2A). Furthermore, the association of TREM2^+^/Iba1^+^ co-expressing cells with 6E10^+^Aβ plaques was also significantly increased in the SCF+G-CSF-treated APP/PS1 mice as compared to the vehicle controls (p<0.05) (Fig. 4D and E) (Supplementary Fig. 2B and Supplementary Fig. 3). These data suggest that the increased TREM2 expression in the Iba1^+^ microglia/macrophages might contribute to the enhancement of Aβ clearance in the brains of aged APP/PS1 mice by SCF+G-CSF treatment.

**Figure 5.**
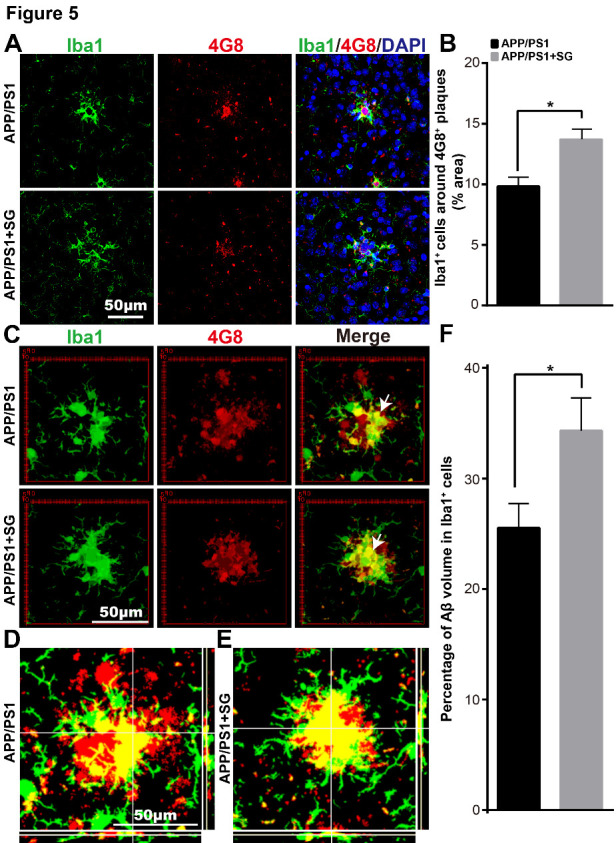
SCF+G-CSF treatment increases the association of Iba1^+^ microglia/macrophages with senile plaques and enhances uptake of 4G8^+^ Aβ by the Iba1^+^ microglia/macrophages in the brains of aged APP/PS1 mice. (A) Representative confocal images illustrate the association of Iba1^+^ cells (green) with 4G8^+^ Aβ plaques (red) in the brains of aged APP/PS1 mice. Blue: Nuclear counterstaining by DAPI. (B) Quantification of the percentage of Iba1^+^ area in/surrounding the 4G8^+^ Aβ plaques in the brains of aged APP/PS1 mice with or without SCF+G-CSF treatment. (C) Representative 3-dimensional projected images reveal the overlapped (yellow) Iba1^+^ cells (green) with 4G8^+^ Aβ (red) in the brains of aged APP/PS1 mice. The white arrows indicate the co-expression (yellow) of 4G8^+^ Aβ and Iba1^+^ cells in the brains of aged APP/PS1 mice. (D and E) Representative orthographic view of z-stack images (12 z-stacks with 1μm intervals) shows the co-expression (yellow) of 4G8^+^ Aβ (red) and Iba1^+^ cells (green) in the brains of aged APP/PS1 mice. (F) Quantification data present the percentage of 4G8^+^ Aβ volume within the Iba1^+^ cells in the brains of aged APP/PS1 mice treated with or without SCF+G-CSF. N=4-5. Mean ± SEM. *p<0.05 by Student’s t-test.

**Figure 6.**
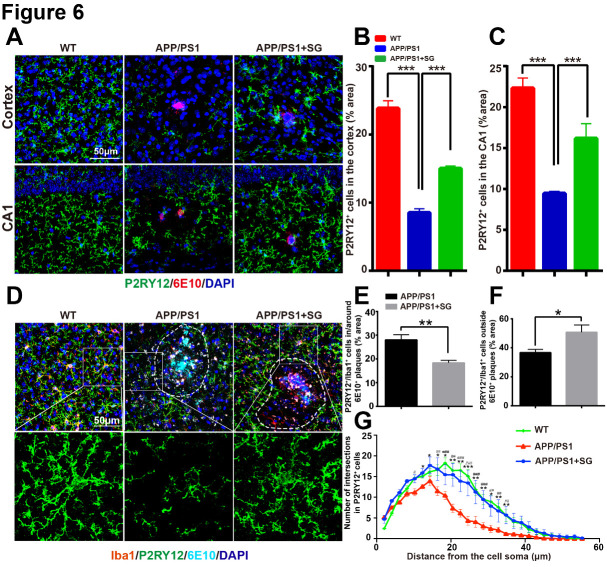
SCF+G-CSF treatment modulates the expression of P2RY12 in the brains of aged APP/PS1 mice. (A) Representative confocal images show P2RY12 (green) and 6E10 (red) double immunofluorescence staining in the cortex and hippocampal CA1 of aged APP/PS1 mice and age-matched wild type (WT) mice. (B and C) Quantification data reveal the percentage of P2RY12^+^ area in the cortex (B) and CA1 region (C) in aged APP/PS1 mice treated with/without SCF+G-CSF and age-matched WT control mice. N=4-5. Mean ± SEM. ***p<0.001, one-way ANOVA followed by Fisher’s LSD *post hoc* test. (D) Representative confocal images illustrate the P2RY12 (green) expressing Iba1^+^ (red) cells within and outside the 6E10^+^ Aβ plaques (cyan) in the brains of aged APP/PS1 mice and age-matched WT mice. Dash line: separate the area of the vicinity and outside of Aβ plaques. (E) Quantification data show the percentage of P2RY12^+^/Iba1^+^co-expressing area within and arround the 6E10^+^ Aβ plaques in the brains of aged APP/PS1 mice treated with or without SCF+G-CSF. (F) Quantification data present the percentage of P2RY12^+^/Iba1^+^co-expressing area outside the 6E10^+^ Aβ plaques (10μm away from the border of Aβ plaques) in the brains of aged APP/PS1 mice treated with or without SCF+G-CSF. N=4-5. Mean ± SEM. *p<0.05, **p<0.01 by Student’s t-test. (G) The number of branches in the P2RY12^+^ microglia outside the 6E10^+^ Aβ plaques is quantified by Sholl analysis. Blue: Nuclear counterstaining by DAPI. N=4-5. Mean ± SEM. APP/PS1 vs. APP/PS1+SG: *p<0.05, **p<0.01, ***p<0.001*;* APP/PS1 vs. WT: *#p*<0.05, *##p*<0.01, *###p*<0.001 *by* One-way ANOVA followed by Fisher’s LSD *post hoc* test.

### SCF plus G-CSF treatment promotes the phagocytosis of Aβ by microglia/macrophages in aged APP/PS1 mice

Next, we sought to examine the effects of SCF+G-CSF treatment in Aβ removal by microglia/macrophages. To this end, we quantified colocalization of Iba1^+^ cells and Aβ plaques and the volume of engulfed Aβ in Iba1^+^ cells in the brains of APP/PS1 mice using immunofluorescence double staining and confocal imaging. As shown in Figure 5A and B, SCF+G-CSF treatment significantly increased the interaction/association of Iba1^+^ cells with 4G8^+^ plaques (p<0.05). Moreover, 3-dimensional (3D) analysis revealed that the volume of 4G8^+^ Aβ within the Iba1^+^ cells was significantly increased by SCF+G-CSF treatment (p<0.05) (Fig. 5C-F).

CD68 is a transmembrane glycoprotein and belongs to the family of lysosome-associated membrane proteins and the family of scavenger receptors [45]. CD68 is mainly expressed in lysosomes of microglia and macrophages [46-48] and plays an important role in phagocytizing Aβ [48]. To further validate our findings in Iba1 expressing cells, we quantified the co-localization of 4G8^+^ Aβ with CD68^+^ compartment. We found that CD68^+^ cells around the 4G8^+^ plaques were significantly increased in the cortex of SCF+G-CSF-treated APP/PS1 mice (p<0.05) (Supplementary Fig. 4A-C). In addition, the colocalization of 4G8^+^ Aβ in CD68^+^ lysosomal compartments was also significantly increased in the cortex of SCF+G-CSF-treated APP/PS1 mice (p<0.05) (Supplementary Fig. 4D).

These findings demonstrate that SCF+G-CSF treatment enhances the accumulation of Iba1^+^ and CD68^+^ microglia/macrophages surrounding the senile plaques and promotes phagocytic clearance of Aβ by the Iba1^+^ and CD68^+^ microglia/macrophages in the brains of aged APP/PS1 mice.

**Figure 7.**
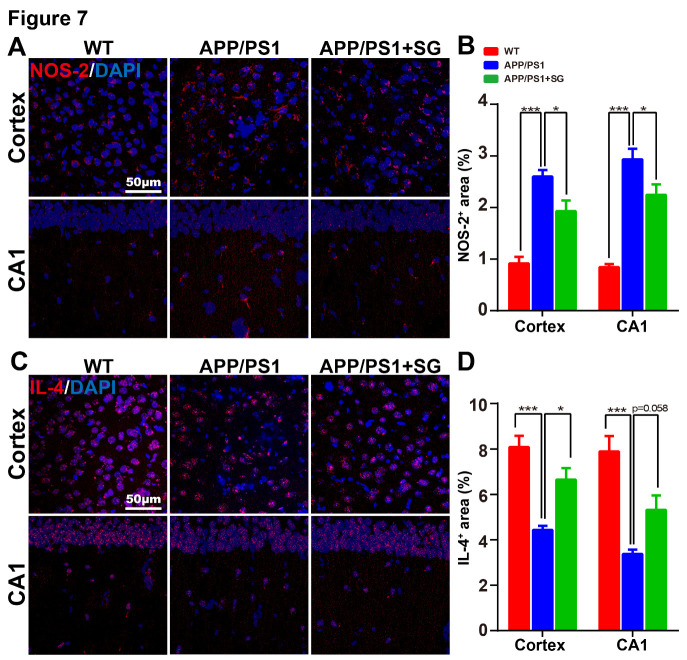
SCF+G-CSF treatment decreases NOS-2 expression and increases IL-4 expression in the brains of aged APP/PS1 mice. (A) Representative confocal images illustrate the NOS-2 immunopositive staining (red) in the cortex and hippocampal CA1 of aged APP/PS1 mice treated with or without SCF+G-CSF and age-matched wild type (WT) mice. (B) Quantification data show the percentage of NOS-2^+^ area in the cortex and CA1 region of aged APP/PS1mice (with or without SCF+G-CSF treatment) and age-matched WT mice. (C) Representative confocal images illustrate the IL-4 immunopositive staining (red) in the cortex and CA1 region of aged APP/PS1 mice treated with or without SCF+G-CSF and age-matched WT mice. (D) Quantification data show the percentage of IL-4^+^ area in the cortex and CA1 of aged APP/PS1 mice treated with or without SCF+G-CSF and age-matched WT control mice. Blue: Nuclear counterstaining by DAPI. N=4-5. Mean ± SEM. *p<0.05, ***p<0.001, one-way ANOVA followed by Fisher’s LSD *post hoc* test.

**Figure 8.**
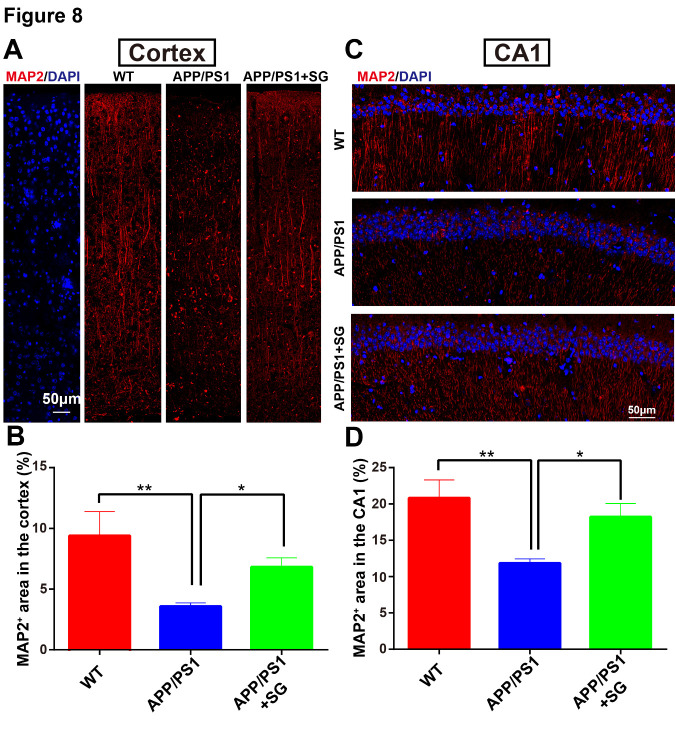
SCF+G-CSF treatment increases dendritic density in the cortex and hippocampus of aged APP/PS1 mice. (A) Representative tile scanning confocal images show MAP2 immunopositive dendrites (red) in the entire cortex of aged APP/PS1 mice treated with/without SCF+G-CSF and age-matched wild type (WT) mice. (B) Quantification data show the changes in the percentage of MAP2^+^ area in the cortex among aged APP/PS1 mice treated with/without SCF+G-CSF and age-matched WT mice. (C) Representative tile scanning confocal images illustrate MAP2 immunopositive dendrites (red) in the CA1 region of aged APP/PS1 mice treated with/without SCF+G-CSF and age-matched WT mice. (D) Quantification data reveal the changes in the percentage of MAP2^+^ area in the CA1 region among aged APP/PS1 mice treated with/without SCF+G-CSF and age-matched WT mice. Blue: Nuclear counterstaining by DAPI. N=4-5. Mean ± SEM. *p<0.05, **p<0.01, one-way ANOVA followed by Fisher’s LSD *post hoc* test.

### SCF plus G-CSF treatment increases cerebral resting microglial cells in aged APP/PS1 mice

Long-term microglial activation-induced persistent inflammation in the brain is a significant pathological feature in AD [49, 50]. P2RY12 is a unique and specific marker expressed in non-activated microglia (also known as homeostatic microglia or resting microglia) that can distinguish the microglial cells from peripheral monocytes/macrophages [51]. Emerging evidence demonstrates that loss/reduction of P2RY12 expression is linked to microglial activation and neuroinflammation [52, 53]. To identify the interaction between Aβ removal and inflammatory status after SCF+G-CSF treatment, we quantified P2RY12 expressing homeostatic microglia in the brains of aged APP/PS1 mice. Our data showed that the P2RY12 expression was significantly decreased in the cortex (p<0.001) and hippocampal CA1 (p<0.001) of aged APP/PS1 mice as compared to the WT controls (Fig. 6A-C). Importantly, the reduced P2RY12 expression in the brains of aged APP/PS1 mice was significantly elevated by SCF+G-CSF treatment in both the cortex (p<0.001) and CA1 (p<0.001) (Fig. 6A-C). Interestingly, further analysis revealed that the P2RY12^+^/Iba1^+^ resting microglial cells immediately next to the Aβ plaques were significantly decreased in the brains of SCF+G-CSF-treated APP/PS1 mice as compared to the vehicle controls (p<0.01) (Fig. 6D, E). By contrast, the P2RY12^+^/Iba1^+^ resting microglial cells at a distance of 10μm away from the border of Aβ plaques were significantly increased by SCF+G-CSF treatment (p<0.05) (Fig. 6F). Since the resting microglial cells are characterized by typical small cell bodies with long and thin processes containing multiple branches [54, 55], Sholl analysis was used to quantify the branches of the P2RY12^+^ microglia. The data of Sholl analysis demonstrated that the branches of the P2RY12^+^ microglia were significantly decreased in the brains of APP/PS1 mice as compared to the WT controls (p<0.05). SCF+G-CSF treatment significantly increased the branches of the P2RY12^+^ microglia in the brains of APP/PS1 mice as compared to the vehicle control APP/PS1 mice (p<0.05) (Fig. 6G).

**Figure 9.**
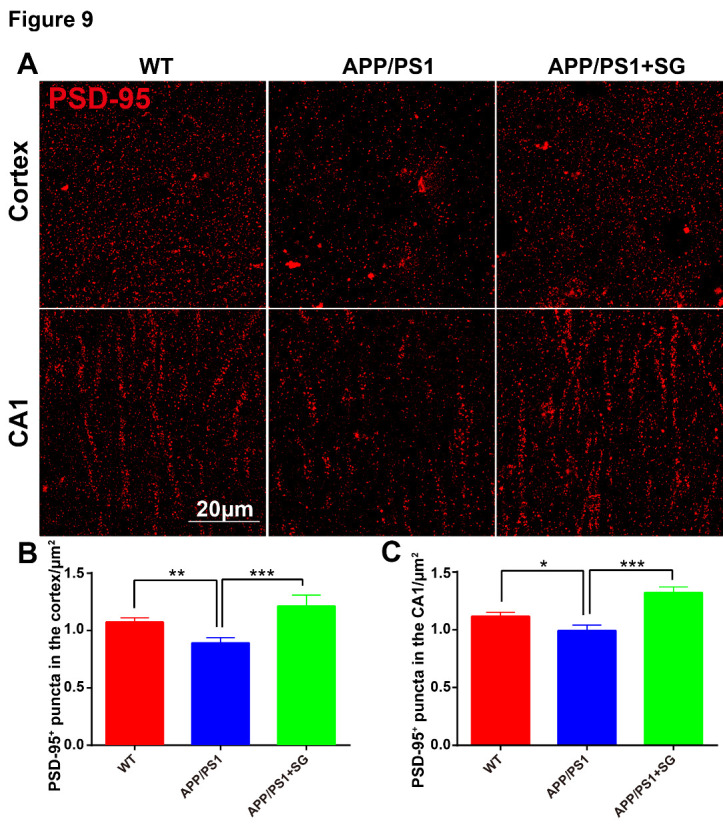
SCF+G-CSF treatment increases PSD-95 positive post-synapses in the cortex and hippocampus of aged APP/PS1 mice. (A) Representative confocal images show PSD-95 immunopositive puncta (red) in the cortex and hippocampal CA1 of aged APP/PS1 mice treated with/without SCF+G-CSF and age-matched wild type (WT) mice. (B and C) Quantification data show the changes of PSD-95^+^ puncta in the cortex (B) and hippocampal CA1 (C) among aged APP/PS1 mice treated with/without SCF+G-CSF and age-matched WT mice. N=4-5. Mean ± SEM. *p<0.05, **p<0.01, ***p<0.001, one-way ANOVA followed by Fisher’s LSD *post hoc* test.

To further verify the contribution of SCF+G-CSF treatment in maintaining/restoring the homeostatic state of microglia, we used another homeostatic microglial marker, TMEM119, to detect the homeostatic microglia. We observed that the expression of TMEM119 in the cortex of APP/PS1 mice was significantly decreased as compared to the WT mice (p<0.001) (Supplementary Fig. 5A, B). SCF+G-CSF- treated APP/PS1 mice showed a trend toward increasing TMEM119 positive microglia in the cortex when compared to the vehicle control APP/PS1 mice (p=0.053) (Supplementary Fig. 5B). In the cortical area at a distance of 15μm away from the border of Aβ plaques, TMEM119 positive microglial cells were significantly increased by SCF+G-CSF treatment (p<0.05) (Supplementary Fig. 5C).

These findings demonstrate that SCF+G-CSF treatment leads to increases in microglial homeostasis in the brains of aged APP/PS1 mice.

### SCF plus G-CSF treatment decreases NOS-2 and increases IL-4 expression in the brains of aged APP/PS1 mice

To further evaluate the effects of SCF+G-CSF intervention on the inflammatory reactivity in the brains of aged APP/PS1 mice, we quantified the expression of pro-inflammation factor, NOS-2, and anti-inflammation factor, IL-4, in the brains of aged APP/PS1 mice through immunohistochemistry. We observed that the expression of NOS-2 was significantly increased in both the cortex (p<0.001) and hippocampal CA1 region (p<0.001) of aged APP/PS1 mice as compare to the WT controls (Fig. 7A, B). SCF+G-CSF treatment significantly decreased the NOS-2 expression in both the cortex (p<0.05) and CA1 region (p<0.05) of aged APP/PS1 mice (Fig. 7A, B). Furthermore, in comparison with the WT controls, the aged APP/PS1 mice in the vehicle control group showed significant decreases of IL-4 expression in both the cortex (p<0.001) and CA1 region (p<0.001) (Fig. 7C and D). In the SCF+G-CSF-treated aged APP/PS1 mice, however, the IL-4 expression was elevated in the cortex (p<0.05) and CA1 region (p=0.058) as compared to the vehicle controls (Fig. 7C and D). These results further confirm the contribution of SCF+G-CSF treatment in modulating the inflammatory status in the brains of aged male APP/PS1 mice.

**Figure 10.**
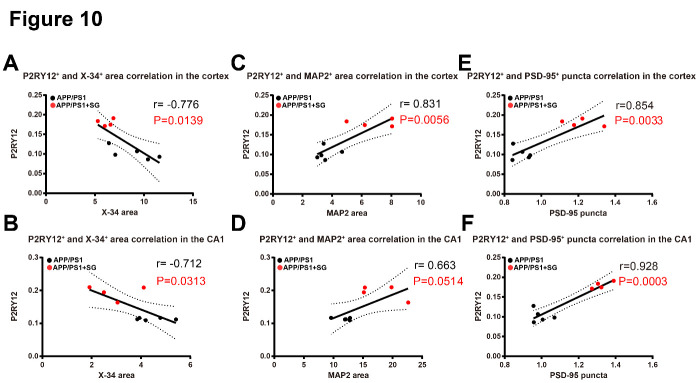
Correlation between Aβ plaques, homeostatic microglia, dendrites and synapses in the brains of aged APP/PS1 mice treated with or without SCF+G-CSF. (A and B) P2RY12^+^ homeostatic microglia show a significantly negative correlation with X-34^+^ fibrillar Aβ plaques in the cortex (r = -0.776, p<0.05) (A) and hippocampal CA1 (r = -0.712, p<0.05) (B). Note that increased P2RY12^+^ resting microglia are correlated with the reduced X-34^+^ fibrillar Aβ plaques in the cortex and hippocampal CA1 in the SCF+G-CSF-treated aged APP/PS1 mice. (C and D) P2RY12^+^ microglia display a positive correlation with MAP2^+^ dendrites in the cortex (r=0.831, p<0.01) (C) and hippocampal CA1 (r=0.663, p=0.051) (D). Note that increased P2RY12^+^ homeostatic microglia are correlated with the increased MAP2^+^ dendrites in the cortex and CA1 in the SCF+G-CSF-treated aged APP/PS1 mice. (E and F) P2RY12^+^ microglia show a significantly positive correlation with PSD-95^+^ puncta in the cortex (r=0.854, p<0.01) (E) and hippocampal CA1 (r=0.928, p<0.001) (F). Note that the increased P2RY12^+^ homeostatic microglia are correlated with the increased PSD-95^+^ puncta in the cortex and CA1 in the SCF+G-CSF-treated aged APP/PS1 mice.

To explore the cell type expression of NOS-2 and IL-4 in the WT and APP/PS1 mice, we performed double immunofluorescence staining of NOS-2 and IL-4 with NeuN (neuronal marker), Iba1 (microglial marker) and GFAP (astrocyte marker). We observed that NOS-2 was expressed in the neurons (NeuN^+^) (Supplementary Fig. 6A) and microglia (Iba1^+^) (Supplementary Fig. 6B) in both aged WT and APP/PS1 mice, but no obvious expression was seen in the astrocytes (GFAP^+^) (Supplementary Fig. 6C). IL-4 was expressed in the neurons (NeuN^+^) (Supplementary Fig. 7A), microglia (Iba1^+^) (Supplementary Fig. 7B) and astrocytes (GFAP^+^) (Supplementary Fig. 7C) in both aged WT and APP/PS1 mice.

### SCF plus G-CSF treatment reverses the loss of cerebral dendrites and synapses in aged APP/PS1 mice

Dendritic degeneration and synaptic loss induced by Aβ neurotoxicity and chronic inflammation are the major causes for the cognitive impairments in AD [56-58]. Building on our findings that SCF+G-CSF treatment enhanced the Aβ removal and ameliorated the inflammatory status, we then sought to determine the efficacy of SCF+G-CSF treatment in remodeling dendrites and synapses in aged APP/PS1 mice. As shown in Figure 8, MAP2^+^ dendrites were significantly decreased in both the entire cortex (p<0.01) (Fig. 8A, B) and hippocampal CA1 region (p<0.01) (Fig. 8C, D) of aged APP/PS1 mice as compared to the WT controls. The reduced MAP2^+^ dendrites in the brains of aged APP/PS1 mice were significantly restored by SCF+G-CSF intervention in both the entire cortex (p<0.05) (Fig. 8A, B) and CA1 region (p<0.05) (Fig. 8C, D). Furthermore, PSD-95 (a post-synaptic marker) positive puncta were significantly decreased in both the cortex (p<0.01) (Fig. 9A, B) and CA1 region (p<0.05) (Fig. 9A, C) of the aged APP/PS1 mice as compared to the WT controls. SCF+G-CSF treatment completely prevented the loss of PSD-95^+^ puncta in both the cortex (p<0.001) (Fig. 9A, B) and CA1 region (p<0.001) (Fig. 9A, C) of aged APP/PS1 mice as compared to the vehicle controls. These data suggest that SCF+G-CSF treatment reverses the loss of cerebral dendrites and synapses in aged APP/PS1.

A recent study has revealed that neurofibrillary pathology occurs in the brain of 12-24-month-old APPswe/PS1dE9 (APP/PS1) mice [59]. To determine the effects of SCF+G-CSF treatment on tau pathology in aged APP/PS1 mice, we used the AT8, a tau phosphorylation (Ser202, Thr205) antibody, to probe tangle-like structures in 26~27-month-old APP/PS1 mice. We observed apparent aggregated AT8^+^ tau pathological structures in the cortex of APP/PS1 mice (Supplementary Fig. 8A). The number of tau pathological structures (AT8^+^ puncta) was not significantly different between SCF+G-CSF-treated and vehicle control APP/PS1 mice (Supplementary Fig. 8B and C). However, SCF+G-CSF-treated APP/PS1 mice showed significant reductions in the average size of AT8^+^ puncta in the cortex as compared to the vehicle control APP/PS1 mice (p<0.001) (Supplementary Fig. 8D). This observation indicates that SCF+G-CSF treatment ameliorates tau pathology in aged APP/PS1 mice.

### Correlation between Aβ plaques, homeostatic microglia, dendrites and synapses

To further determine the interaction between Aβ plaque load and the quantity of resting microglia, dendrites and synapses, we performed a correlation analysis in both the vehicle controls and SCF+G-CSF-treated aged APP/PS1 mice. The data revealed that a significantly negative correlation was seen between P2RY12^+^ resting microglia and X-34^+^ fibrillar Aβ plaques in both the cortex (r = -0.776, p<0.05) (Fig. 10A) and hippocampal CA1 (r = -0.712, p<0.05) (Fig. 10B) of the APP/PS1 mice. These data suggest that the areas with high Aβ plaque deposits show less expression of P2RY12^+^ resting microglia, while the areas with reduced Aβ plaque deposits display increased P2RY12^+^ resting microglia. This observation indicates that Aβ deposits lead to loss of P2RY12^+^ resting microglia.

However, a positive correlation was found between P2RY12^+^microglia and MAP2^+^ dendrites in the cortex (r=0.831, p<0.01) (Fig. 10C) and hippocampal CA1 (r=0.663, p=0.0541) of the APP/PS1 mice (Fig. 10D). Furthermore, there was a significantly positive correlation between P2RY12^+^microglia and PSD-95^+^puncta in both the cortex (r=0.854, p<0.01) (Fig. 10E) and hippocampal CA1 (r=0.928, p<0.001) (Fig. 10F) of the APP/PS1 mice. These findings demonstrate that the microenvironment with increased P2RY12^+^resting microglia/homeostatic microglia is beneficial to maintain dendrites and synapses in the brain of aged APP/PS1 mice.

## DISCUSSION

Using a widely-used mouse model of cerebral amyloidosis in AD research, the present study has demonstrated the therapeutic efficacy of combined SCF and G-CSF treatment in aged male APP/PS1 mice. Our data have revealed that SCF+G-CSF treatment reduces diffuse and fibrillar Aβ deposits, increases the association of microglia/macrophages with senile plaques, and enhances Aβ uptake by microglia/macrophages in the brains of aged APP/PS1 mice. Furthermore, SCF+G-CSF treatment increases homeostatic microglia and ameliorates inflammatory status in the brains of aged APP/PS1 mice. Systemic administration of SCF+G-CSF also reverses the loss of dendrites and synapses and reduces pathological tau in the brains of aged APP/PS1 mice. These data suggest that SCF+G-CSF treatment ameliorates the pathological severity in the brain of aged APP/PS1 mice.

Microglia are the resident macrophages in the central nervous system. Microglial cells constantly survey their local microenvironment, control neurogenesis and synaptic generation, and contribute to the maintenance of brain homeostasis. Microglia also detect the first signs of pathogenic invasion and tissue damage and support tissue repair [60]. Iba1 is the marker to detect both resting and active microglia in the brains of AD patients and mouse models of AD [32, 61, 62]. Many other markers have been used to identify specific phenotypes of microglia. Recent transcriptomic studies have revealed that homeostatic microglia gradually adopt a unique phenotype of phagocytic disease-associated microglia (DAM) in the brains of amyloidogenic mouse models and AD patients [62-64]. Progressive Aβ accumulation accelerates DAM formation [64]. DAM phenotype is characterized by downregulation of key homeostatic genes including P2ry12, Cx3cr1 and Tmem119 and upregulation of AD-associated genes (e.g. Trem2, ApoE, CD68) [62, 63]. The location of DAM is revealed in the vicinity of the Aβ plaques in both mouse models and AD patients [62, 63, 65, 66]. Whether the activated microglia (DAM) play a beneficial role, detrimental role or both in AD progression remains to be clarified [67]. It is also not clear the underlying mechanism of microglial phenotype switch during disease progression and why DAM clusters concentrate in the area of Aβ plaques. Emerging evidence shows that fibrillar Aβ is the main driver for switching microglia to DAM phenotype [68]. Deleting ApoE or Trem2 blocks microglial accumulation around Aβ plaques [64, 69]. It has been shown that DAM around Aβ plaques are phagocytic cells to phagocytize Aβ [62]. These findings suggest that DAM accumulation in the area of Aβ plaques may be involved in limiting Aβ accumulation.

Hematopoietic growth factors have been demonstrated to effectively reduce Aβ load in the brains of APP/PS1 mice. In addition to SCF and G-CSF, macrophage colony-stimulating factor (M-CSF) and granulocyte-macrophage colony-stimulating factor (GM-CSF) are also the family members of hematopoietic growth factors. Systemic injection of M-CSF [70] or GM-CSF [71] to APP/PS1 mice results in decreases of cerebral Aβ deposits. An *in vitro* study reveals that M-CSF treatment increases microglial proliferation and phagocytosis of Aβ in primary cultured adult human microglia [72]. In a previous study, we have demonstrated that subcutaneous administration of SCF+G-CSF in 9-month-old APP/PS1 mice results in reduced Aβ load at the age of 18 months, indicating a long-term beneficial effect of SCF+G-CSF in inhibiting Aβ accumulation [32]. It has been reported that subcutaneous injections of G-CSF alone in 7-9 or 13-15 month-old APP/PS1 mice lead to Aβ reduction in 3 weeks [73], while clinical evidence reveals no effect on reducing Aβ load by G-CSF alone treatment [74]. In a long-term study, we have observed that SCF+G-CSF combination treatment in 10-month-old APP/PS1 mice shows more effective and stable effects in reducing Aβ load as compared to G-CSF or SCF alone (Li et al., unpublished observations). In addition to the beneficial effects observed in middle-aged APP/PS1mice, the present study has revealed that subcutaneous administration of SCF+G-CSF at an elderly age (~25 months old) reduces the burden of both fibrillar Aβ and diffuse Aβ in the APP/PS1 mice.

Although the precise mechanism underlying the SCF+G-CSF-reduced Aβ load remains unclear, our data suggest that increasing phagocytic removal of Aβ by microglia/macrophages is involved in the SCF+G-CSF-reduced Aβ load in the brain of aged APP/PS1 mice. Both resident Iba1^+^ microglia and bone marrow-derived Iba1^+^ macrophages have been proposed to be involved in the clearance of Aβ [32, 72, 75, 76]. As it is hard to unambiguously distinguish these cellular populations, their individual contribution to Aβ clearance in AD is not entirely clear. In the present study, we have revealed that the engulfed 4G8^+^ Aβ in the Iba1^+^ microglia/macrophages is increased by SCF+G-CSF treatment. CD68 is the marker for detecting lysosomes of microglia and macrophages [46-48]. Our data have also demonstrated that the colocalization of 4G8^+^ Aβ in CD68^+^lysosomes of microglia/macrophages is increased by SCF+G-CSF treatment in aged APP/PS1 mice. These findings suggest that SCF+G-CSF treatment-reduced Aβ load in the brains of aged APP/PS1 mice is modulated by enhanced Aβ uptake and degradation (Aβ clearance) through phagocytosis by microglia/macrophages. Similar findings have been reported in the M-CSF study showing that Iba1^+^ microglia-internalized Aβ in the brain of APP/PS1 mice is increased by systemic injection of M-CSF [70]. An *in vitro* study also demonstrates the efficacy of M-CSF in enhancing the ability of microglia to phagocytize and degrade fibrillar Aβ [76].

Recent studies have demonstrated that TREM2 plays a vital role in mediating phagocytosis and degradation of Aβ by Iba1^+^ microglia/macrophages in transgenic mouse models of AD [43, 44]. Loss of function mutation in TREM2 (p.R47H) has been shown to be associated with an increased risk of AD [77, 78]. It has been documented that TREM2 plays a critical role in governing microglial cell activation, driving DAM phenotype, and promoting microglial cell accumulation around Aβ plaques [69, 79]. Our data have revealed that TREM2 is mainly expressed in the Iba1^+^ microglia/macrophages surrounding the Aβ plaques, while TREM2 could not be detected in the Iba1^+^ cells distant from the plaques in the brains of APP/PS1 mice. This observation is in line with the findings reported by other investigators [80]. The findings of the present study have also uncovered that SCF+G-CSF treatment increases TREM2 expression in the Iba1^+^ microglia/macrophages surrounding the senile plaques, suggesting that TREM2 upregulation is involved in SCF+G-CSF-enhanced Aβ clearance by the Iba1^+^ microglia/macrophages in the senile plaques. In addition to SCF+G-CSF-increased TREM2 expression in the Iba1^+^ microglia/macrophages surrounding the Aβ plaques and SCF+G-CSF-increased CD68-expressing microglia/macrophages around the Aβ plaques, it is worth noting that SCF+G-CSF treatment also leads to reduced P2RY12^+^/Iba1^+^ homeostatic microglia in the vicinity of the Aβ plaques. Our observations suggest that SCF+G-CSF treatment in aged APP/PS1 mice enhances the transformation of homeostatic microglia into phagocytic DAM phenotype in the area next to the Aβ plaques. This SCF+G-CSF-enhanced microglial phenotype transformation occurring particularly at the location surrounding the Aβ plaques may be necessary to reinforce Aβ removal and restrict Aβ-induced pathology. A recent transcriptomic study has shown that the population of DAM contains both pro-inflammatory DAM and anti-inflammatory DAM. The anti-inflammatory DAM express phagocytic genes. Using a pharmacological approach to promote anti-inflammatory DAM or inhibit pro-inflammatory DAM leads to enhanced Aβ clearance in 5xFAD mice [81]. It would be an interesting question to be addressed in future studies whether promoting anti-inflammatory DAM transformation is involved in the SCF+G-CSF-enhanced Aβ clearance in the brains of aged APP/PS1 mice.

As stated earlier, resident microglia play a vital role in the maintenance of brain homeostasis. Losing the homeostatic phenotype in microglia is thought to be linked to pathological severity in the context of AD [65, 67]. P2RY12 is a specific marker for detecting the homeostatic/resting microglia in the brain [51, 53, 82]. P2RY12 is also a purinergic receptor which plays a key role in maintaining homeostatic microglia [67]. In addition to accumulation of DAM around the amyloid plaques, reduced P2RY12^+^ microglia have been found in the cerebral parenchyma in mouse models and AD patients [66, 67]. In agreement with these findings reported by others, the data of the present study have revealed a large reduction of P2RY12^+^ microglia in both the cortex and hippocampus of aged APP/PS1 mice. While the consequences of P2RY12 downregulation in AD are not fully understood, P2RY12 expression in microglia has been proposed to have protective effects in the context of AD [67]. In the cerebral cortex of AD patients, reductions of P2RY12 immunoreactivity are correlated with neuropathological scores and synaptic loss [83]. In mouse models of neurodegenerative diseases including AD, P2RY12 immunoreactivity in the brain is reduced at onset and disease peak but restored during recovery [65]. These findings suggest that P2RY12 expression levels in cerebral microglia serve as a biomarker to evaluate pathological severity in the context of AD. This view is further supported by our findings showing that the P2RY12 expression levels are negatively correlated with fibrillar Aβ deposition (X34 positive plaques) but positively correlated with MAP2^+^ dendritic density and PSD-95^+^post-synaptic puncta in the brains of aged APP/PS1 mice. Notably, SCF+G-CSF treatment increases the P2RY12^+^microglia in the cortex and hippocampus, and augments P2RY12^+^/Iba1^+^ cells distant from the Aβ plaque regions in aged APP/PS1 mice. Our findings indicate that SCF+G-CSF treatment improves the recovery of homeostatic phenotype of microglia and ameliorates pathological severity in the brains of aged APP/PS1 mice. The SCF+G-CSF treatment-enhanced recovery of homeostatic microglia in the brains of aged APP/PS1 mice is further supported by our findings in analyses of TMEM119 immunoreactivity and the branches in P2RY12^+^microglia. The reduced TMEM119^+^homeostatic microglia and decreased branches of the P2RY12^+^ homeostatic microglia in the cortex of aged APP/PS1 mice are reversed by SCF+G-CSF treatment. It requires further examinations to elucidate how SCF+G-CSF treatment enhances the recovery of homeostatic microglia in the brain of aged APP/PS1 mice. We propose that interventional molecules/chemicals targeting Aβ clearance and modulating microglial transition into homeostatic phenotype would be helpful therapeutic approaches to restrict pathological progression in AD.

Microglial activation has been thought to be essential for the clearance of accumulated Aβ in the brains of transgenic mouse models of AD [84, 85]. However, chronically activated microglia continuously secrete pro-inflammatory mediators, leading to persistent neuroinflammation in AD. The inflammatory events may compromise the beneficial role of microglia on phagocytosis and degradation of Aβ [49, 86]. Therefore, an ideal therapeutic approach for AD would target not only the enhancement of Aβ removal but also inhibition of neuroinflammation. Neurotoxicity caused by elevated inflammatory response plays a crucial role in the progression of AD [87, 88]. It has been evidenced that IL-4, an anti-inflammatory cytokine, ameliorates Aβ-induced neuroinflammation [89]. NOS-2 is a pro-inflammatory enzyme producing the inflammatory mediator nitric oxide [90]. Increased expression of NOS-2 is involved in the neuronal damage induced by Aβ injection [91]. We have observed that both IL-4 and NOS-2 are expressed in cerebral neurons and microglia. In addition, SCF+G-CSF treatment decreases the expression of NOS-2 and increases the levels of IL-4 in the brains of APP/PS1 mice. This observation demonstrates that SCF+G-CSF treatment reduces neuroinflammation in the brains of aged APP/PS1 mice.

It has been documented that Aβ accumulation and chronic neuroinflammation-induced dendritic loss play a vital role in cognition impairments in APP/PS1 mice [56, 57, 92-94]. MAP2 is a neuron-specific cytoskeletal protein that is enriched in the dendrites. MAP2 has been shown to be crucially involved in determining and stabilizing dendritic shape [95]. MAP2 expression is decreased in 6 or 8-month-old APP/PS1 mice as compared to the age-matched controls [96, 97]. PSD-95, a post-synaptic marker, is essential for maintaining the function of the synapse. Reduced PSD-95 is also seen in the 8 or 12-month-old APP/PS1 mice [98, 99]. In line with these studies, we have observed that both the MAP2^+^ dendrites and PSD-95^+^ post-synapses are decreased in 26-27-month-old APP/PS1 mice as compared to age-matched WT controls. SCF+G-CSF treatment ameliorates the loss of MAP2^+^ dendrites and PSD-95^+^ synapses in the cortex and hippocampal CA1 of aged APP/PS1 mice. The precise mechanism underlying the SCF+G-CSF-increased dendrites and synapses in aged APP/PS1 mice remains unknown. SCF+G-CSF treatment in aged APP/PS1 mice enhances Aβ removal and reduces neuroinflammation, suggesting that the SCF+G-CSF-ameliorated pathological severity in the brain of aged APP/PS1 mice may be beneficial to the maintenance of dendrites and synapses. In addition, our previous study has demonstrated a direct effect of SCF and G-CSF in supporting neurite extension. SCF+G-CSF synergistically enhances neurite extension and neural network structure formation in cultured primary cortical neurons [26]. In a mouse model of chronic stroke, we have also observed that SCF+G-CSF treatment increases MAP2^+^ dendrites and PSD-95^+^ synapses in the ipsilesional cortex [28]. Thus, SCF+G-CSF may restrict dendritic and synaptic loss by promoting the growth of dendrites and synapses in the brains of aged APP/PS1 mice. Furthermore, a study reported by Biscaro and co-workers shows that inhibition of microglial activation protects hippocampal neurogenesis in APP/PS1 mice [100]. On the other hand, accumulating evidence reveals that microglia are essentially involved in synaptic circuit formation and refinement, as well as synaptic excitability and function in the physiologic state of the developing or the adult brain [101-104]. In the present study, we have observed that the P2RY12^+^ homeostatic microglial area is positively correlated with the MAP2^+^ dendritic area and PSD-95^+^ synaptic puncta, indicating that the SCF+G-CSF-increased resting microglia and SCF+G-CSF-improved homeostasis of brain microenvironment may play supportive and reparative roles in maintaining dendrites and synapses in the brains of aged APP/PS1 mice. Finally, intracellular pathological tau aggregation in neurons has been shown to accelerate post-synapse loss in the dendrites [105]. Aβ interaction with pathological tau has recently been proposed as a major pathomechanism in AD [106, 107]. Spontaneous accumulation of tau aggregates has been observed in the brain of aged APP/PS1 mice [59]. Our findings reveal that the size of aggregated tau (AT8^+^ puncta) in the cortex of aged APP/PS1 mice is reduced by SCF+G-CSF treatment. Further studies are needed, however, to explore how SCF+G-CSF treatment in aged APP/PS1 mice restricts pathological tau aggregation.

The limitations of the present study are the lack of a biochemistry approach to validate our observations in immunohistochemistry and the lack of a control group with SCF+G-CSF treatment in age-matched WT mice to determine whether the SCF+G-CSF-ameliorated neuroinflammation and synaptic loss are the results of decreasing Aβ load.

In total, the present study provides the evidence that SCF+G-CSF treatment increases Aβ clearance, promotes recovery of homeostatic microglia, decreases inflammation, reduces aggregated tau, and restricts the loss of dendrites and synapses in the brains of aged male APP/PS1 transgenic mice. SCF+G-CSF-enhanced DAM accumulation surrounding the Aβ plaques for removing pathological Aβ, together with the SCF+G-CSF-enhanced recovery of homeostatic microglia distant from the Aβ plaques for maintaining dendrites and synapses are the unique modulation processes to restrict AD pathology and promote brain repair in the context of AD. Our results indicate that the combination of two hematopoietic growth factors, SCF and G-CSF, may have therapeutic potential for AD, even at the late-stage.

## Supplementary Materials

The Supplemenantry data can be found online at: www.aginganddisease.org/EN/10.14336/AD.2020.0201.
