# Supplementary material for: Reparative Effects of Stem Cell Factor and Granulocyte Colony-Stimulating Factor in Aged APP/PS1 Mice
Source: Aging Dis. 2020 Dec 1;11(6):1423–43. doi: 10.14336/AD.2020.0201 (PMC7673847; doi:10.14336/AD.2020.0201)
Supplement: Supplementary file 1 — The Supplemenantry data can be found online at: www.aginganddisease.org/EN/10.14336/AD.2020.0201. [file AD-11-6-1423-s.pdf]

SUPPLEMENTARY DATA

Reparative Effects of Stem Cell Factor and Granulocyte Colony-Stimulating Factor in Aged APP/PS1 Mice

Xingzhi Guo[1], Yanying Liu[1], David Morgan[2], Li-Ru Zhao[1*]

© 2020. Guo X et al. Published online at http://www.aginganddisease.org/EN/10.14336/AD.2019.0201

SUPPLEMENTARY DATA

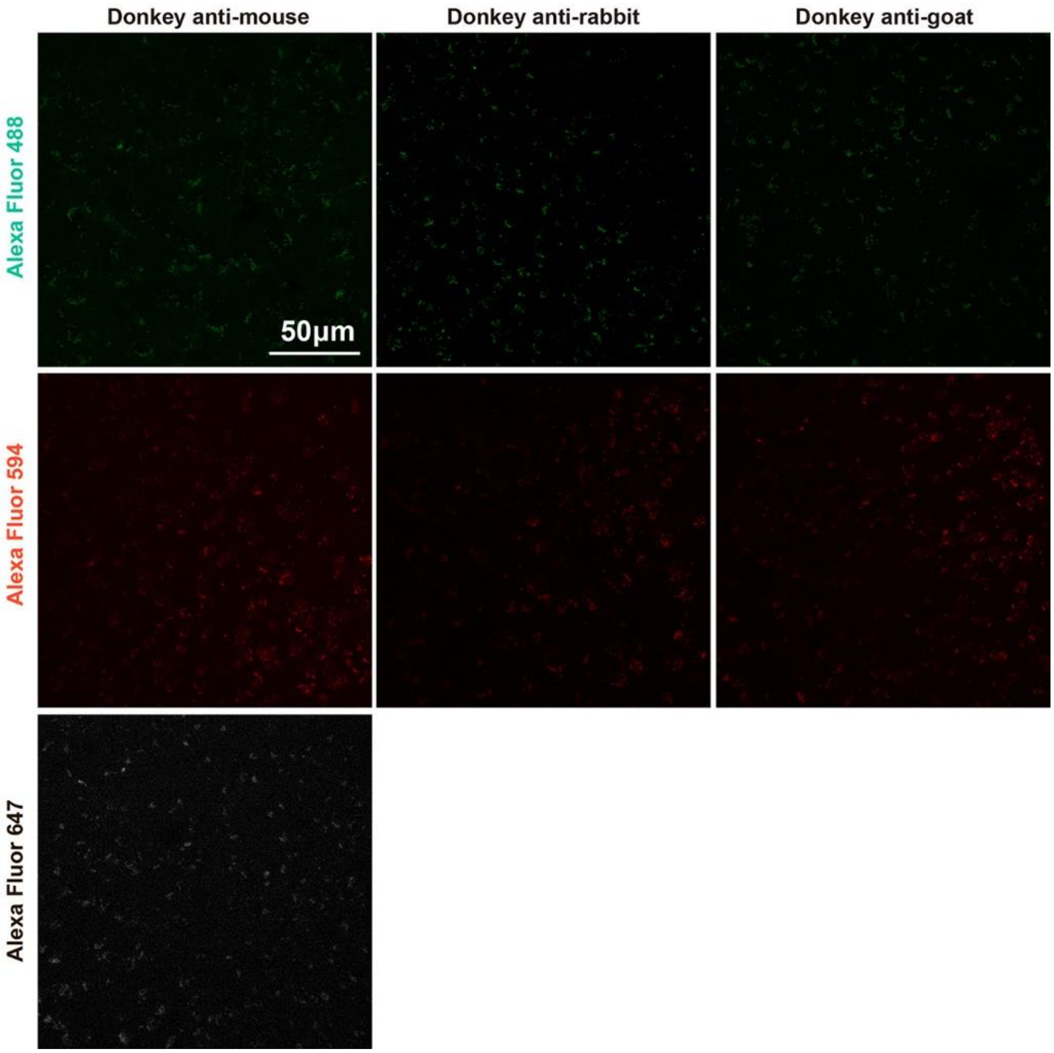

Supplementary Figure 1. **Representative images show the buffer controls for the secondary antibodies used in this study. The** background fluorescence is subtracted from immunofluorescence positive staining in data analysis using ImageJ.

© 2020. Guo X et al. Published online at http://www.aginganddisease.org/EN/10.14336/AD.2019.0201

SUPPLEMENTARY DATA

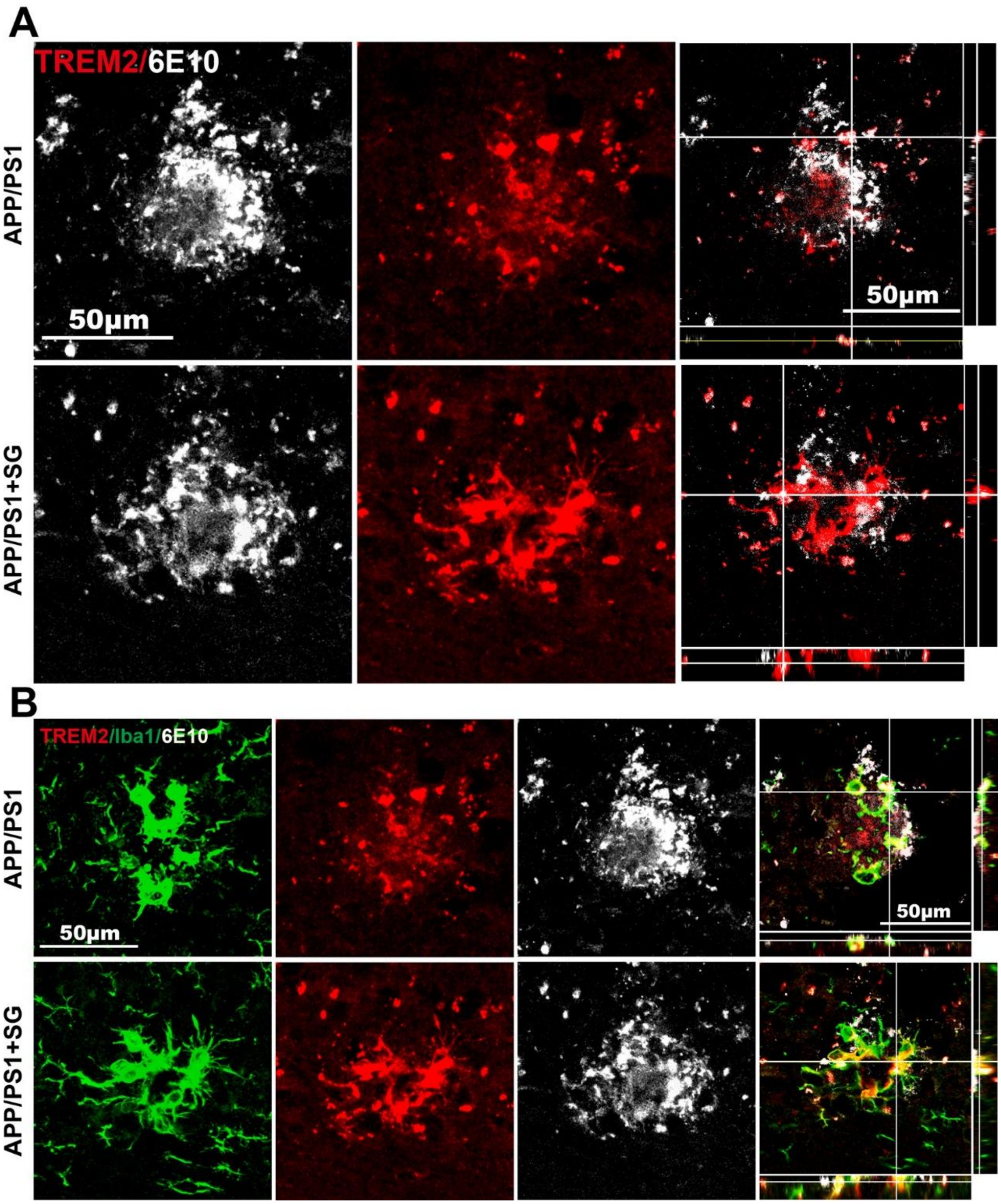

Supplementary Figure 2. Representative orthographic view of z-stack images (12 z-stacks with 1μm intervals) with single color plane illustrates the colocalized TREM2 (red) and 6E10 (white) in the brains of aged APP/PS1 mice. (**A**). Representative orthographic view of z-stack images (12 z-stacks with 1μm intervals) with single color plane displays the co-expression (yellow) of TREM2 (red) and Iba1 (green) around the 6E10^{+} plaques in the brains of APP/PS1 mice (**B**).

© 2020. Guo X et al. Published online at http://www.aginganddisease.org/EN/10.14336/AD.2019.0201

SUPPLEMENTARY DATA

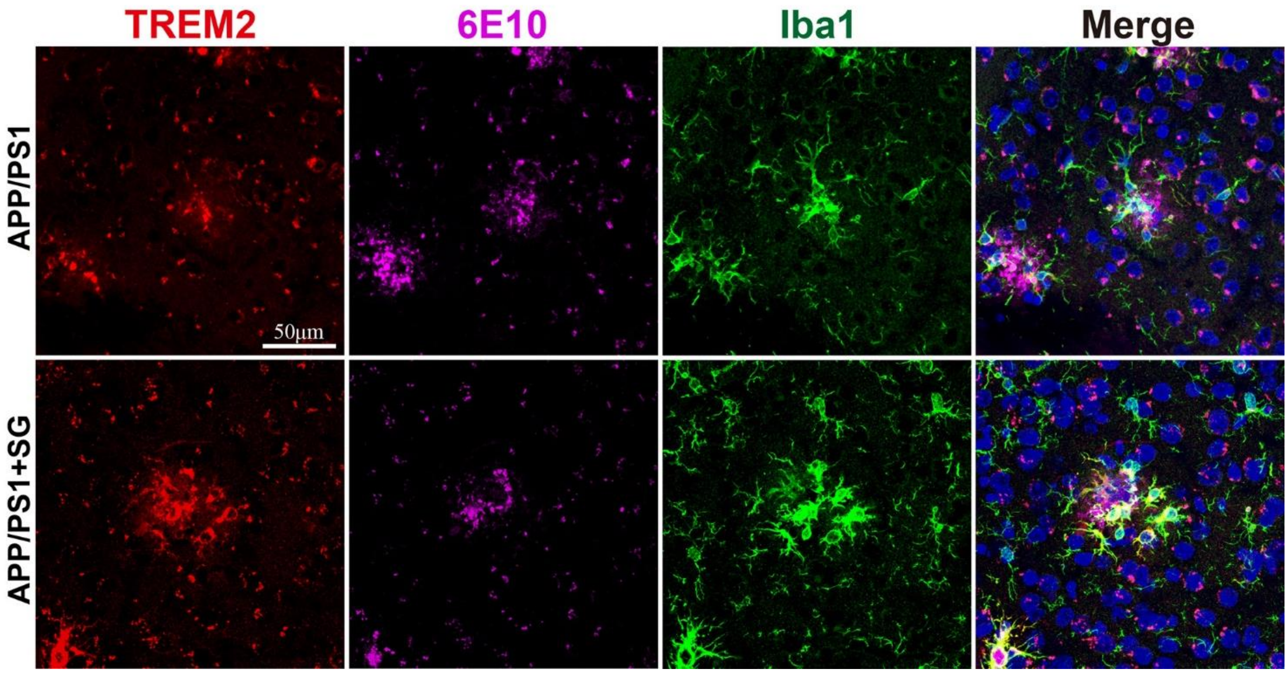

Supplementary Figure 3. Representative confocal images of TREM2 (red), 6E10 (purple) and Iba1 (green) triple immunofluorescence staining in the brains of aged APP/PS1 mice with or without SCF+G-CSF treatment. Blue: Nuclear counterstaining by DAPI.

© 2020. Guo X et al. Published online at http://www.aginganddisease.org/EN/10.14336/AD.2019.0201

SUPPLEMENTARY DATA

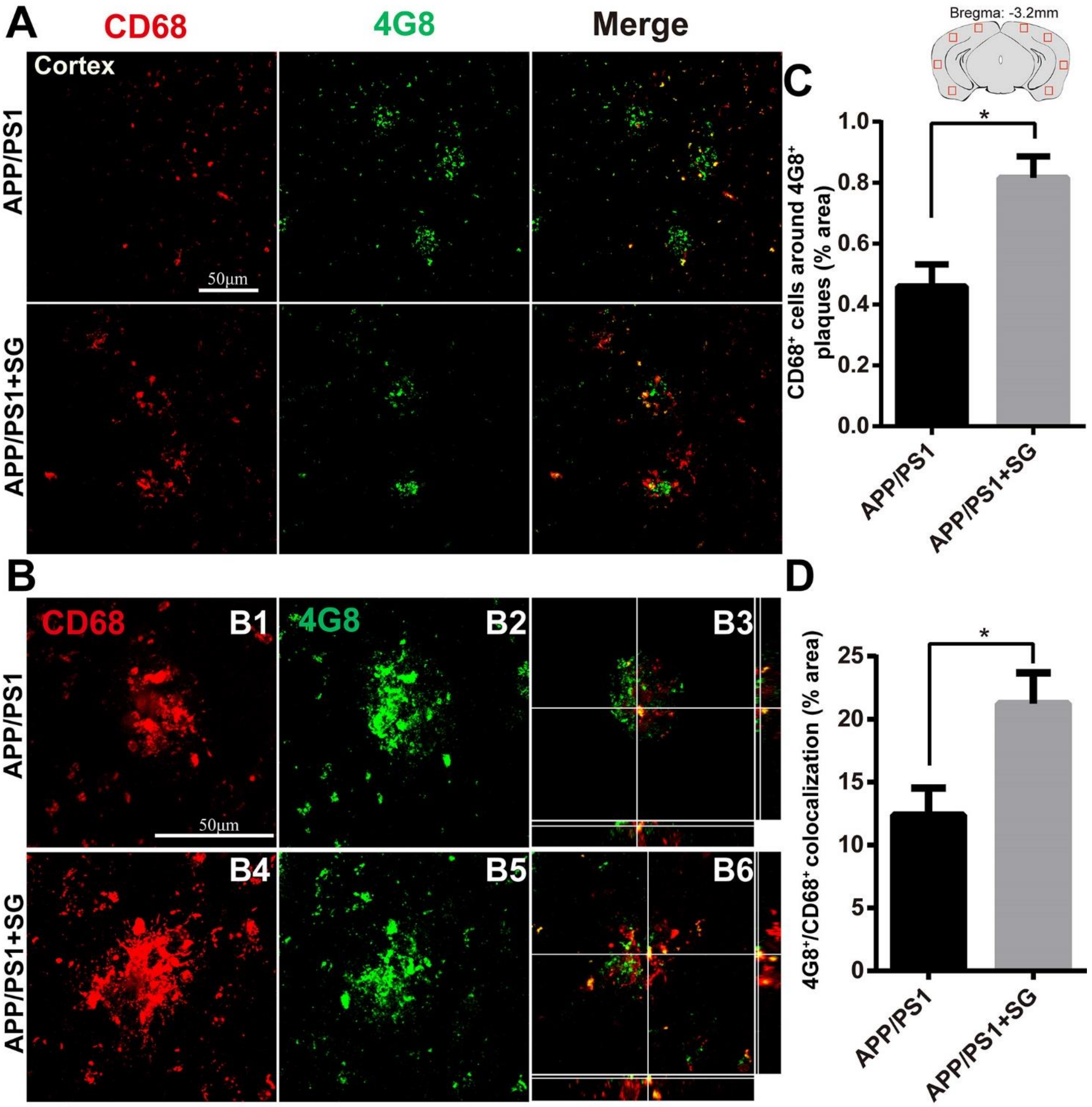

Supplementary Figure 4. SCF+G-CSF treatment increases CD68 expressing cells surrounding the 4G8+ senile plaques and enhances phagocytic clearance of 4G8+ Aβ by CD68+ microglia/macrophages. (**A**) Representative confocal images of CD68 (red) and 4G8 (green) double immunofluorescence staining in the brains of aged APP/PS1 mice. (**B**) Representative orthographic view of z-stack images (12 z-stacks with 1μm intervals) illustrates the colocalized CD68+ lysosomal compartments (red) and 4G8+ Aβ (green) in the brains of aged APP/PS1 mice. B1-2 and B4-5 are the images after z-project using Image J, and B3 and B6 are the single layer of z-stacks. (**C**) Quantification data show the percentage of CD68+ expression area in the vicinity of Aβ plaques (within 10μm from the border of the Aβ plaques) in the brains of aged APP/PS1 mice with or without SCF+G-CSF treatment. (**D**) Quantification data show the percentage of 4G8+ area in the CD68+ lysosomal compartments in the vicinity of Aβ plaques in the brains of aged APP/PS1 mice with or without SCF+G-CSF treatment. N=4-5. Mean ± SEM. * p<0.05 by Student's t-test. Insert: A mouse brain diagram shows where the images are taken (red box: imaging area).

SUPPLEMENTARY DATA

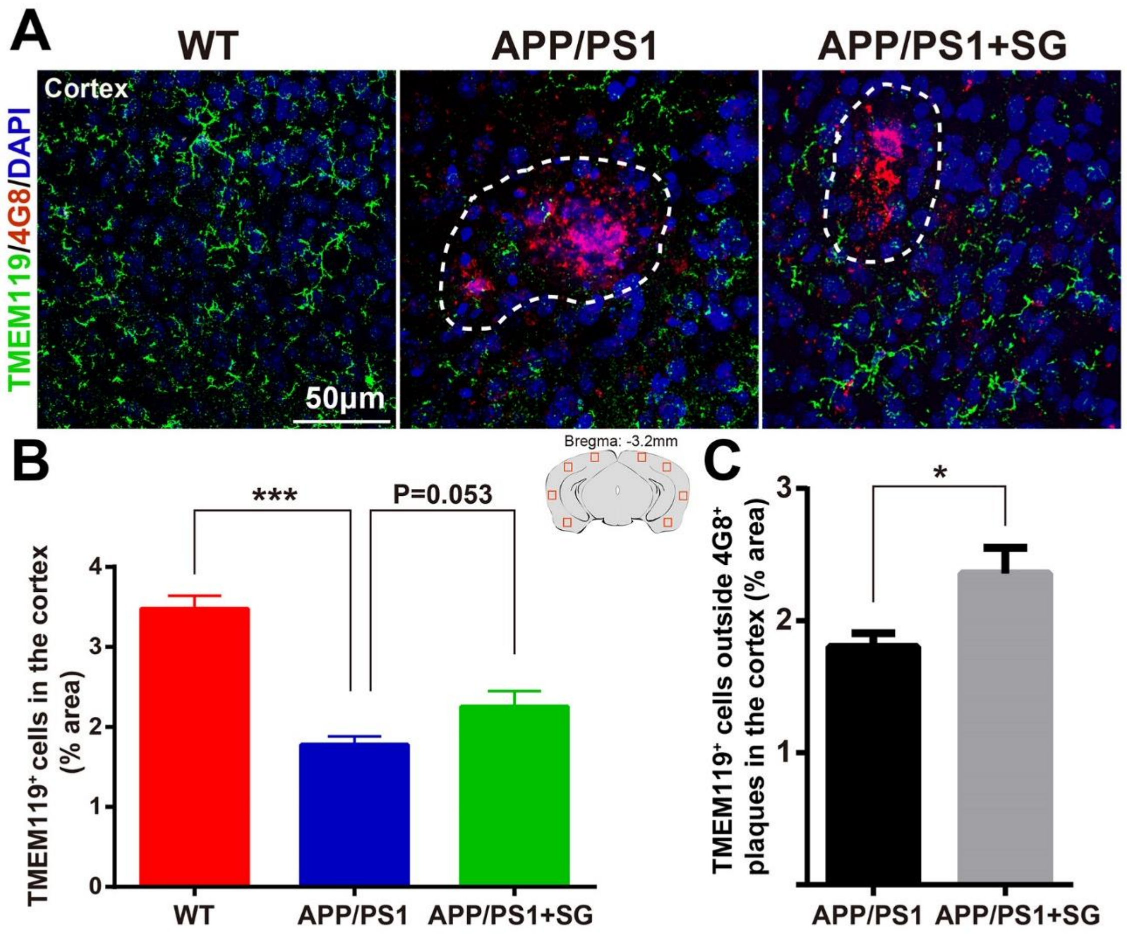

Supplementary Figure 5. **SCF+G-CSF treatment increases TMEM119$^+$ homeostatic microglia in the brains of aged APP/PS1 mice.** (**A**) Representative confocal images of TMEM119 (green) and 4G8 (red) double immunofluorescence staining in the cortex of aged APP/PS1 mice and age-matched wild type (WT) mice. (**B**) Quantification data reveal the percentage of TMEM119$^+$ area in the cortex of aged APP/PS1 mice (with/without SCF+G-CSF treatment) and age-matched WT control mice. N=4-5. Mean ± SEM. ****p*<0.001, by one-way ANOVA followed by Fisher's LSD *post hoc* test. (**C**) Quantification data present the percentage of TMEM119$^+$ area outside the 4G8$^+$ Aβ plaques (15μm away from the border of Aβ plaques) in the brains of aged APP/PS1 mice treated with or without SCF+G-CSF. N=4-5. Mean ± SEM. **p*<0.05 by Student's t-test. Blue: Nuclear counterstaining by DAPI. Dash line: separate the area of the vicinity and outside of Aβ plaques. Insert: A mouse brain diagram shows where the images are taken (red box: imaging area).

© 2020. Guo X et al. Published online at http://www.aginganddisease.org/EN/10.14336/AD.2019.0201

SUPPLEMENTARY DATA

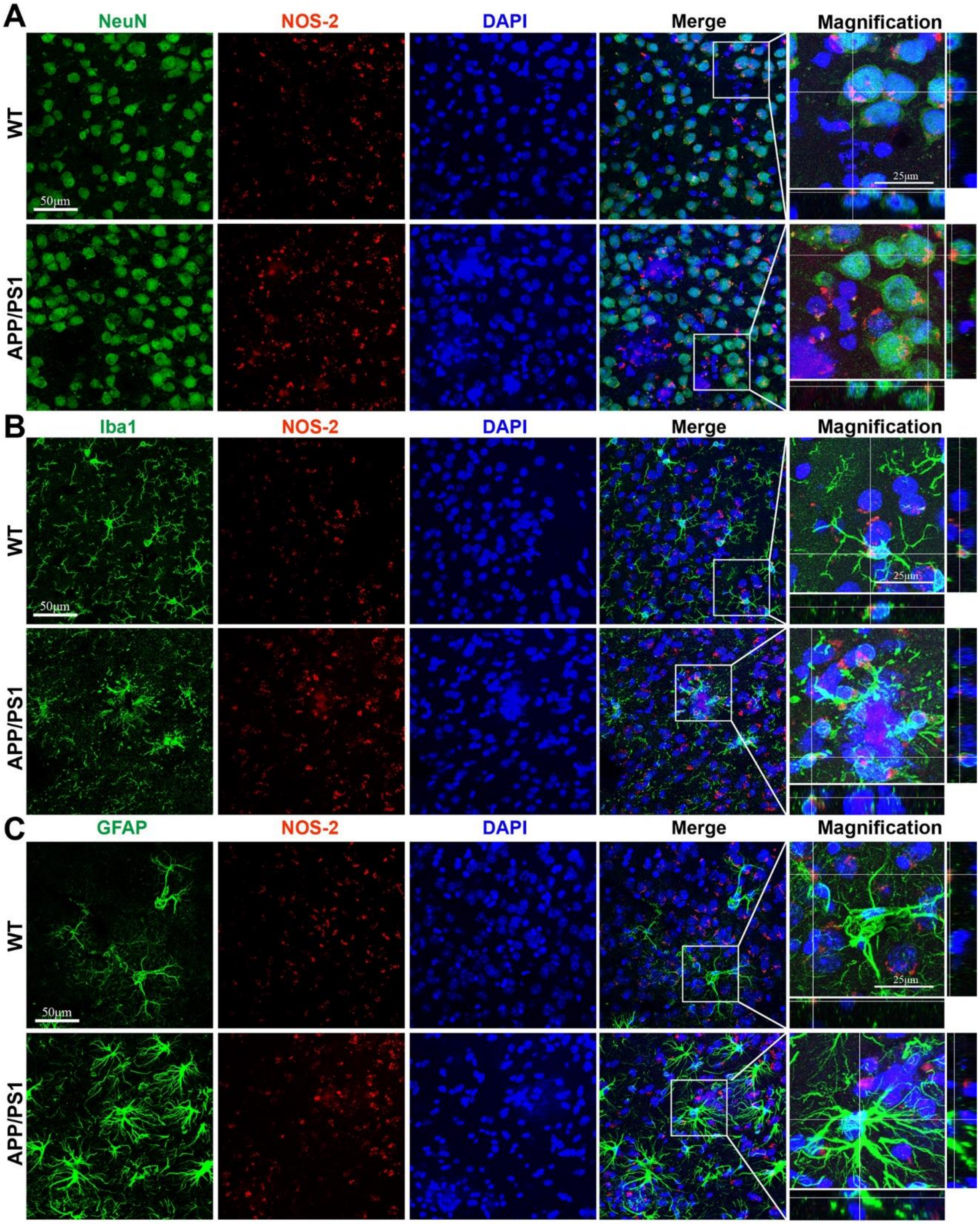

Supplementary Figure 6. **The expression of NOS-2 in NeuN$^+$ neurons and Iba1$^+$ microglia but not in GFAP$^+$ astrocytes in the brains of aged wild type (WT) and APP/PS1 mice.** (**A**) Representative confocal images show the double immunofluorescence staining of NOS-2 and NeuN in the brains of WT and APP/PS1 mice. (**B**) Representative confocal images illustrate the double immunofluorescence staining of NOS-2 and Iba1 in the brains of WT and APP/PS1 mice. (**C**) Representative confocal images for double immunofluorescence staining of NOS-2 and GFAP in the brains of WT and APP/PS1 mice.

© 2020. Guo X et al. Published online at http://www.aginganddisease.org/EN/10.14336/AD.2019.0201

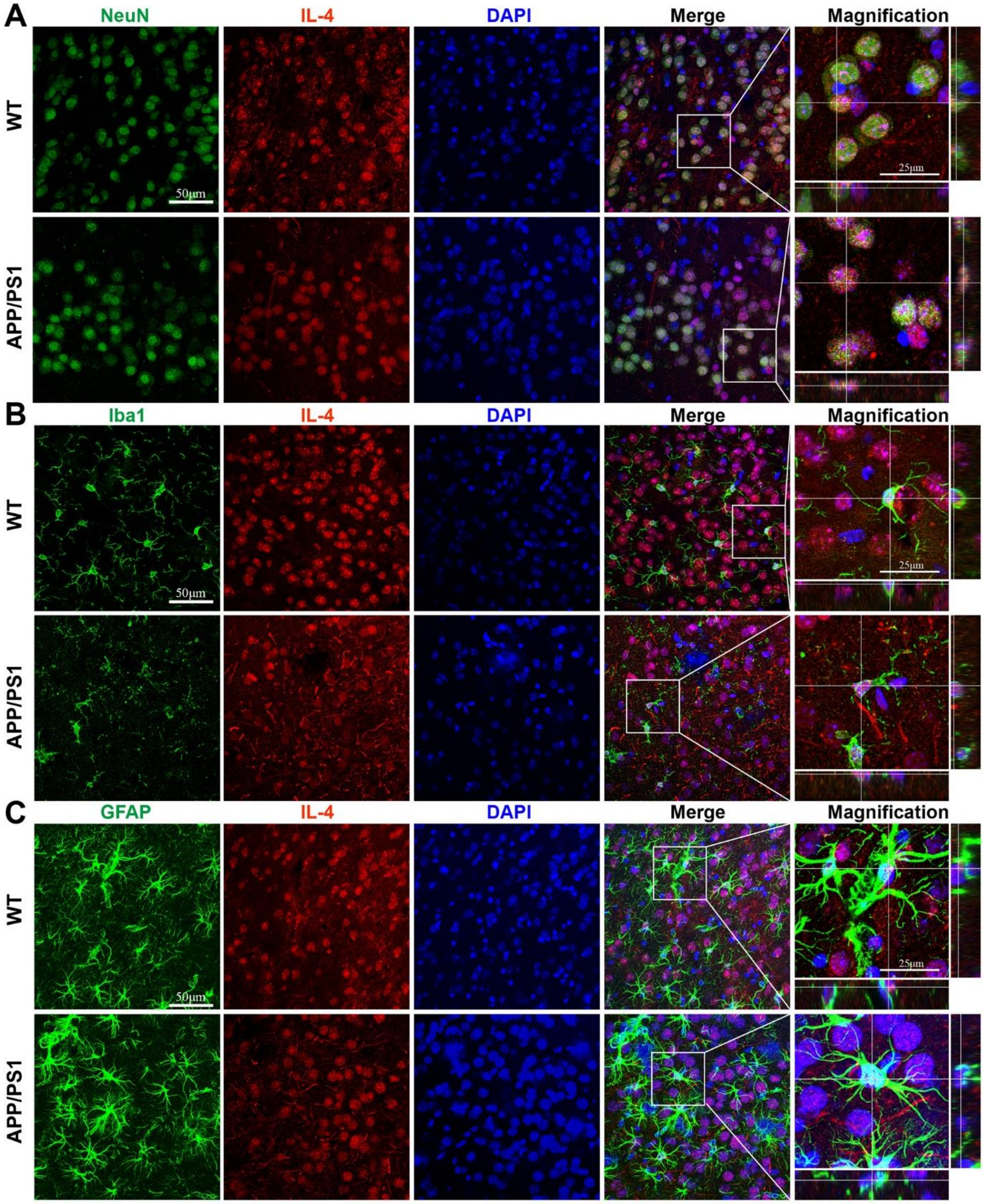

Supplementary Figure 7. The expression of IL-4 in NeuN⁺ neurons, Iba1⁺ microglia and GFAP⁺ astrocytes in the brains of aged wild type (WT) and APP/PS1 mice. (**A**) Representative confocal images show the double immunofluorescence staining of IL-4 and NeuN in the brains of WT and APP/PS1 mice. (**B**) Representative confocal images illustrate the double immunofluorescence staining of IL-4 and Iba1 in the brains of WT and APP/PS1 mice. (**C**) Representative confocal images display the double immunofluorescence staining of IL-4 and GFAP in the brains of WT and APP/PS1 mice.

SUPPLEMENTARY DATA

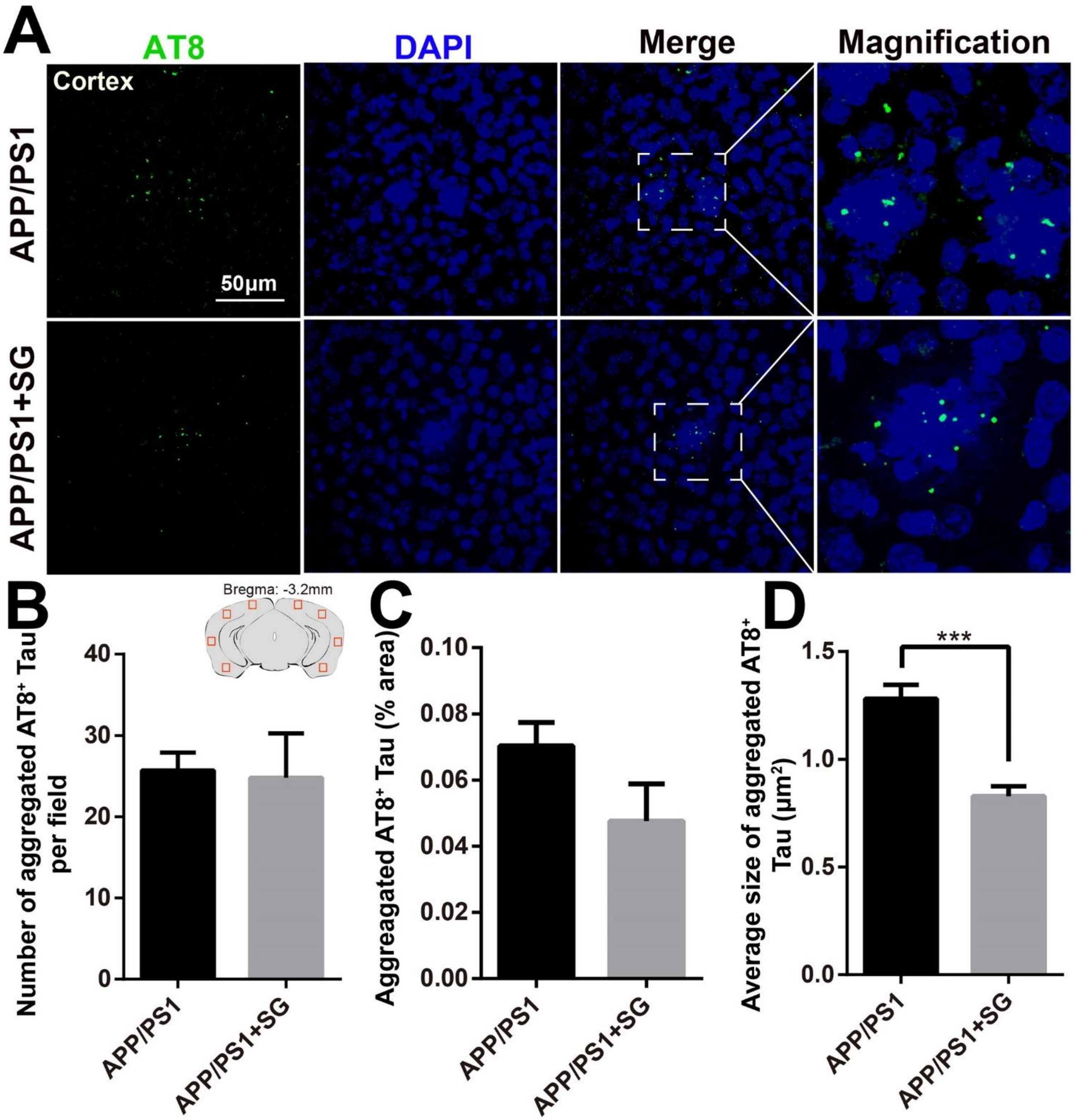

Supplementary Figure 8. **SCF+G-CSF treatment reduces aggregated AT8^{+} tau pathological structures in the brains of aged APP/PS1 mice.** (**A**) Representative confocal images illustrate aggregated AT8^{+} (green) tau pathological structures in the cortex of aged APP/PS1 mice treated with or without SCF+G-CSF. (**B-D**) Quantification data show the number (B), the percentage area (C), and the average size of aggregated AT8^{+} tau pathological structures (D) in the cortex of aged APP/PS1 male mice treated with or without SCF+G-CSF. Blue: Nuclear counterstaining by 4',6-diamidino-2-phenylindole (DAPI). SG: SCF+G-CSF. N=4-5. Mean ± SEM. ****p*<0.001 by Student's t-test. Insert: A mouse brain diagram shows where the images are taken (red box: imaging area).

© 2020. Guo X et al. Published online at http://www.aginganddisease.org/EN/10.14336/AD.2019.0201
